# Mutated Isocitrate Dehydrogenase (mIDH) as Target for PET Imaging in Gliomas

**DOI:** 10.3390/molecules28072890

**Published:** 2023-03-23

**Authors:** Felix Neumaier, Boris D. Zlatopolskiy, Bernd Neumaier

**Affiliations:** 1 Forschungszentrum Jülich GmbH, Institute of Neuroscience and Medicine, Nuclear Chemistry (INM-5), Wilhelm-Johnen-Str., 52428 Jülich, Germany; 2 Institute of Radiochemistry and Experimental Molecular Imaging, Faculty of Medicine and University Hospital Cologne, University of Cologne, Kerpener Str. 62, 50937 Cologne, Germany

**Keywords:** mutated isocitrate dehydrogenase (mIDH), molecular imaging, positron emission tomography (PET), glioma, fluorine-18, radiotracer

## Abstract

Gliomas are the most common primary brain tumors in adults. A diffuse infiltrative growth pattern and high resistance to therapy make them largely incurable, but there are significant differences in the prognosis of patients with different subtypes of glioma. Mutations in isocitrate dehydrogenase (IDH) have been recognized as an important biomarker for glioma classification and a potential therapeutic target. However, current clinical methods for detecting mutated IDH (mIDH) require invasive tissue sampling and cannot be used for follow-up examinations or longitudinal studies. PET imaging could be a promising approach for non-invasive assessment of the IDH status in gliomas, owing to the availability of various mIDH-selective inhibitors as potential leads for the development of PET tracers. In the present review, we summarize the rationale for the development of mIDH-selective PET probes, describe their potential applications beyond the assessment of the IDH status and highlight potential challenges that may complicate tracer development. In addition, we compile the major chemical classes of mIDH-selective inhibitors that have been described to date and briefly consider possible strategies for radiolabeling of the most promising candidates. Where available, we also summarize previous studies with radiolabeled analogs of mIDH inhibitors and assess their suitability for PET imaging in gliomas.

## 1. Introduction

With an annual incidence rate of approximately five to six per 100.000, adult-type diffuse gliomas are the most common malignant tumors of the central nervous system (CNS) [1]. These tumors are thought to arise from glial-like precursor cells harboring oncogenic mutations. They have a diffuse, infiltrative growth pattern and are highly resistant to therapy, making them largely incurable. However, there are significant differences in the prognosis of patients with different subtypes of diffuse glioma, with 5-year survival rates ranging from <5% for the most aggressive forms to 80% for less aggressive subtypes [1]. Mutations in the metabolic enzyme isocitrate dehydrogenase (IDH) are an important biomarker for glioma classification and a potential target for new therapeutic approaches. However, mutated IDH (mIDH) isoforms are so far almost exclusively detected by immunohistochemistry and genomic sequencing, which require invasive tissue sampling and cannot be used for follow-up examinations or longitudinal studies. Positron emission tomography (PET) is a well-established molecular imaging technique based on bioactive compounds labeled with positron-emitting radionuclides, which can be detected non-invasively by measuring the positron-electron annihilation radiation. PET imaging could represent a promising approach for non-invasive assessment of the IDH status in gliomas. However, the development of mIDH-selective PET tracers based on existing inhibitors is associated with several challenges, which include differences in the design criteria for therapeutic drugs and imaging probes as well as the need for structural modifications without adverse effects on the pharmacokinetic properties. The present review illustrates the potential advantages of mIDH-selective PET tracers and highlights possible challenges associated with their development. Subsequently, the main chemical classes of mIDH-selective inhibitors are compiled, their suitability for the development of PET tracers for glioma imaging is discussed, and possible strategies for radiolabeling of the best candidates based on their structure–activity relationship (SAR) are considered. Additionally, the results of previous studies with radiolabeled analogs are described, with a special focus on whether they could potentially be used for PET imaging in glioma.

## 2. Oncogenic IDH Mutations and Their Implications for Gliomagenesis

**Figure 1:**
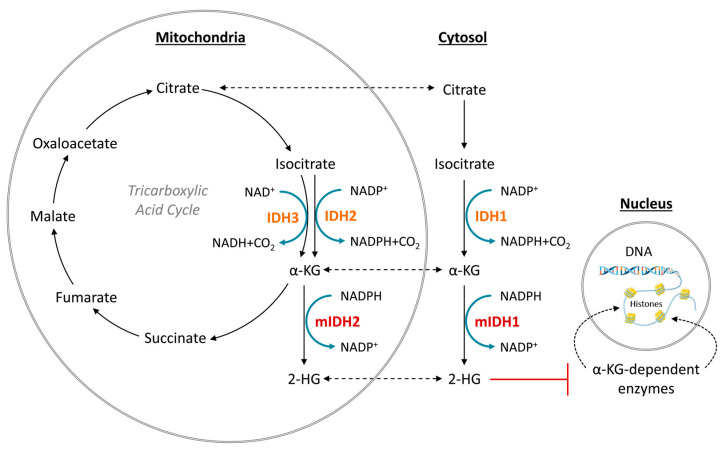
Function of normal (orange) and mutated (red) isocitrate dehydrogenase (IDH) isoforms. Redrawn and modified from [15].

**Figure 2:**
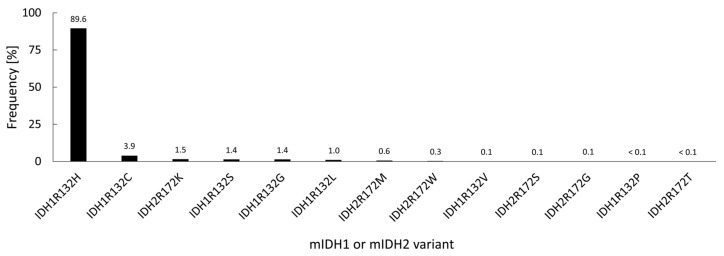
Relative frequency of different IDH mutations in 3489 IDH-mutated gliomas from various studies, calculated based on the data compiled in [3].

Under normal conditions, IDH1 and IDH2 are homodimeric enzymes that catalyze the reversible NADP^+^- and Mg^2+^-dependent decarboxylation of isocitrate to α-ketoglutarate (α-KG) with concomitant production of NADPH, either in the cytosol and peroxisomes (IDH1) or in the mitochondria (IDH2) (Figure 1) [2]. A similar but non-reversible and NAD^+^-dependent reaction in the tricarboxylic acid cycle is catalyzed by the more distantly related heterotetrameric enzyme IDH3, but no mutations of IDH3 in gliomas have been described so far [2]. Instead, the vast majority of known IDH mutations in gliomas are heterozygous missense mutations at codon R132 of IDH1 (>90%) or, much less commonly, at the synonymous codon R172 of IDH2 (<3%), which result in substitution of a highly conserved arginine residue involved in substrate coordination at the active site [2,3,4]. The archetype of these mutations is an arginine-to-histidine substitution in IDH1 (R132H) present in roughly 90% of IDH-mutant gliomas. Non-canonical mutations of IDH1 or IDH2 involving replacement of the corresponding arginine residue by other amino acids (cysteine, serine, valine, glycine, leucine or glutamine) are much less frequent (Figure 2) [3]. A common feature of all these mutations is that they interfere with the normal conversion of isocitrate to α-KG and endow the enzyme with a neomorphic activity that results in NADPH-dependent reduction of α-KG to the potential oncometabolite 2-hydroxyglutarate (2-HG) [5,6]. As a consequence, 2-HG concentrations in IDH-mutated cells are typically >100-fold higher than the concentrations observed in normal cells, which is thought to result in 2-HG-induced competitive suppression of several α-KG-dependent enzymes responsible for epigenetic regulation (Figure 1) [5,7,8]. This, in turn, promotes various epigenetic changes, such as histone and DNA hypermethylation, which may drive the malignant transformation of affected cells by predisposing them to further mutations [8]. Consistent with this notion, IDH mutations in gliomas have been shown to be highly associated with certain other mutations, most notably TP53 mutations or 1q/19q co-deletions, and studies on their timing suggest that they typically precede these mutations [9,10,11,12]. As such, IDH mutations seem to reflect very early events in gliomagenesis that may occur at the tumor precursor stage and are widely considered to be a promising therapeutic target. However, preclinical studies indicate that although mutant IDH1 expression drives malignant transformation, mIDH1 itself may rapidly convert from a driver to a passenger mutation [13]. Accordingly, it is not clear whether IDH mutations remain a viable therapeutic target when more aggressive mutations are acquired in the later stages of the disease. In addition, the metabolic changes associated with IDH mutations have been shown to make tumor cells more sensitive to other therapeutic interventions, which could be exploited for alternative treatment strategies [14]. Nevertheless, the early occurrence of IDH mutations during gliomagenesis means that their expression in IDH-mutated gliomas should be homogenous among tumor cells, making them particularly promising targets for tumor imaging. Furthermore, as mentioned in the introduction and outlined in more detail in the following section, IDH mutations have been recognized as key molecular markers of certain gliomas that distinguish them from other, more malignant forms, so that their non-invasive detection could facilitate glioma classification and therapy planning in affected patients.

## 3. Role of IDH Mutations for Glioma Classification

**Figure 3:**
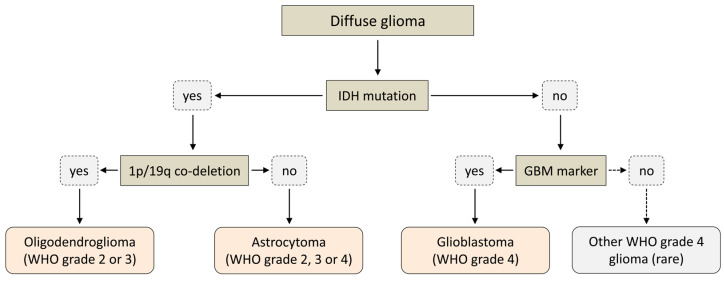
Simplified scheme illustrating the 2021 WHO classification for adult-type diffuse glioma.

Historically, gliomas have been classified according to the presumed cells of origin (e.g., astrocytoma, oligodendroglioma, etc.). Based on certain morphological features (mitotic activity, anaplastic nuclear features, microvascular proliferation and necrosis), they were further assigned to one of four WHO grades, with less malignant lesions corresponding to WHO grades 1–2 (typically referred to as “low-grade gliomas”) and more malignant lesions corresponding to WHO grades 3–4 (typically referred to as “high-grade gliomas”). The most malignant forms (WHO grade 4) were collectively referred to as glioblastomas and often further subdivided into primary glioblastomas (de novo occurring, rapidly growing tumors without clinical or histological evidence of a lower-grade precursor lesion) and secondary glioblastomas (tumors clearly originating from a lower-grade precursor legion and typically associated with a better prognosis) [16]. Histopathologically, the latter subtypes, in particular, could not be reliably distinguished, but analysis of large glioma cohorts revealed that they might indeed represent quite separate diseases with distinct molecular genetics and clinical behavior [16,17,18]. In particular, more than 65% of low-grade gliomas and more than 85% of secondary glioblastomas but less than 5% of primary glioblastomas were found to harbor IDH mutations [9,19]. In addition, patients with high-grade gliomas harboring an IDH mutation were consistently found to have significantly longer survival times than patients with a comparable IDH wildtype glioma [9,20,21,22]. Ultimately, these and other findings led to the incorporation of the IDH mutation status (and other well-established molecular parameters) into the 2016 edition of the WHO classification for gliomas [23]. The 2021 edition further expanded upon this trend and significantly simplified the terminology by introducing a system where the classification of diffuse gliomas in adults is almost entirely determined by two key molecular features (IDH mutation status and 1p/19q co-deletion status), while grading is determined by joint histopathologic and molecular analysis [24,25,26]. According to this system, adult-type diffuse gliomas can be classified into three main entities, which comprise astrocytomas (IDH-mutated gliomas without 1p/19q co-deletion), oligodendrogliomas (IDH-mutated gliomas with 1p/19q co-deletion) and glioblastomas (gliomas without IDH mutation), with an additional group of very rare IDH wildtype tumors not classified as glioblastomas (Figure 3) [24,25,26]. Depending on additional molecular and histological markers, oligodendrogliomas can be assigned WHO grades 2 or 3, while astrocytomas can be assigned WHO grades 2, 3 or 4 (with grade 4 corresponding to tumors formerly designated as secondary glioblastoma) [24,25,26]. Glioblastomas (and other IDH wildtype tumors) are considered the most aggressive forms of diffuse glioma with very poor prognosis and are always assigned WHO grade 4 (Figure 3) [24,25,26].

## 4. Current Approaches for Assessment of the IDH Status in Glioma

Based on the recognition of IDH mutations as key disease-defining features of astrocytomas and oligodendrogliomas that reliably distinguish them from glioblastomas, assessment of the IDH status has become an integral part of the diagnostic algorithm for glioma classification. To this end, tissue samples obtained by biopsy or during tumor resection are analyzed by immunohistochemistry for the most frequent IDH mutation (IDH1_R132H_), which may be complemented by DNA sequencing to detect other non-canonical mutations in IDH1 or IDH2. However, because these approaches require invasive tissue sampling, they are associated with a high risk of complications (e.g., bleeding, infection or neurological symptoms due to direct damage to brain tissue) and cannot be used for routine follow-up examinations or longitudinal studies. To overcome these limitations, various imaging modalities have been evaluated for their ability to non-invasively assess the IDH mutation status in gliomas. An elegant but technically challenging approach is the quantification of tumoral 2-HG concentrations by magnetic resonance spectroscopy (MRS) [27]. Although a meta-analysis of over 450 patients showed a cumulative sensitivity and specificity of more than 90% [28], more recent studies indicate that MRS may yield false positive results in up to 20% of all patients with glioblastomas [29]. An alternative approach based on evidence for altered intra- and extracellular sodium concentrations in IDH-mutated gliomas, is ^23^Na magnetic resonance imaging (MRI) [30,31]. However, although ^23^Na MRI correlates with the IDH mutation status, this approach has only been assessed in a small number of patients, and its diagnostic accuracy remains to be confirmed. Finally, as summarized in the following section, differences in the uptake of several established PET tracers have been shown to be useful for distinguishing the IDH status in glioma patients [32,33,34,35,36,37].

## 5. PET Imaging and Rationale for Development of mIDH-Selective Tracers

**Figure 4:**
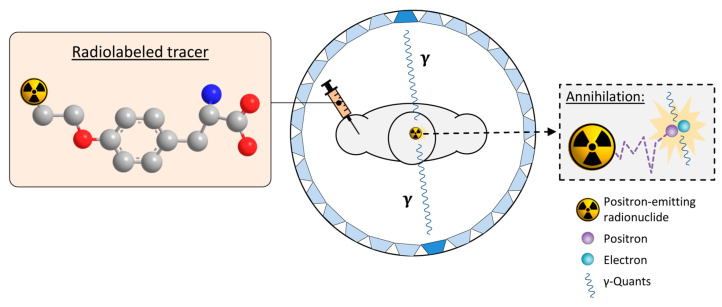
Principle of positron emission tomography (PET) imaging. A tracer labeled with a positron-emitting radionuclide (exemplified by the ^18^F-labeled amino acid *O*-([^18^F]fluoroethyl)tyrosine, with fluorine-18, carbon, nitrogen and oxygen atoms shown as orange, grey, blue or red spheres, respectively while hydrogen atoms are omitted for clarity) is injected into the subject. The subject is placed into the PET-scanner consisting of a ring of opposite detectors (indicated as blue trapezoids). The in vivo biodistribution of the tracer is then tracked by detecting the antiparallel γ-quants produced after the decay of the radionuclide and annihilation of the emitted positrons with electrons in surrounding tissues. Adapted from [45] (CC BY 4.0).

PET imaging is an important nuclear medicine technique that allows for non-invasive visualization and, in some cases, quantification of biochemical processes in vivo using probes labeled with positron-emitting radionuclides (PET tracers). To this end, the subject is administered a suitable PET tracer that preferentially accumulates (unchanged or in the form of metabolites) in the target structures or tissues. The positrons released during the decay of the radionuclide travel a short distance in surrounding tissues before they undergo annihilation with electrons. Annihilation produces a pair of antiparallel 511 keV γ-quants that can be detected by opposite detectors of the ring-shaped PET scanner within a very short timeframe (several ns) [38,39,40] (Figure 4). Since the location of the decay must be in close proximity to the line connecting the two detectors (coincidence line), a three-dimensional image of the tracer distribution can be reconstructed from the corresponding coincidence lines after a sufficient number of decays has been collected [38,40]. Typically, very low radiotracer amounts are applied for PET imaging so that there are no pharmacodynamic effects on the underlying metabolic processes and the radiation dose to the subject remains low. Although various radionuclides can be used for PET imaging, labeling of small molecules for preclinical and clinical applications is most frequently performed with carbon-11 (^11^C: t_1/2_ ≈ 20 min) or fluorine-18 (^18^F: t_1/2_ ≈ 110 min), which can be produced in no-carrier-added form (n.c.a.) in high activity amounts using a cyclotron. An advantage of carbon-11 is that it can be introduced into any organic molecule without structural modification. Furthermore, the short half-life of ^11^C enables multiple PET scans in a subject on the same day. However, the short half-life also severely limits the applicable methods for ^11^C-labeling and impedes the commercialization of ^11^C-labeled tracers. In contrast, the longer half-life of fluorine-18 enables more elaborate labeling chemistry and tracer distribution to several PET centers from the same radiopharmaceutical unit (“satellite” approach), making it the preferred radionuclide for preclinical and clinical PET imaging. Various ^18^F-labeled tracers that visualize tumor-associated changes in glucose metabolism, amino acid transport or other processes are routinely used or have been evaluated for the diagnosis, prognostication, and monitoring of gliomas [41,42]. As already mentioned, some of these tracers have also shown some promise in predicting the presence of IDH mutations in gliomas. Most notably, static and dynamic *O*-([^18^F]fluoroethyl)tyrosine]([^18^F]FET) uptake parameters, as well as [^18^F]FET radiomics, have been used to distinguish the IDH status in glioma patients, with good diagnostic accuracy in most but not all studies [31,32,33,34,35]. In addition, preclinical or clinical findings indicate that differences in the uptake of other established tracers, such as (2-[^18^F]fluoroethyl)choline, 6-[^18^F]fluoro-3,4-dihydroxyphenylalanine ([^18^F]FDOPA) or [^18^F]DPA-714, could be used to infer the IDH mutation status in gliomas [36,37]. However, a common drawback of all existing PET tracers is that they can only provide indirect evidence for the presence of mIDH. In contrast, PET imaging with mIDH-selective tracers would enable the direct detection of mutated IDH proteins, facilitating reliable glioma classification and differential diagnosis in the case of ambiguous brain lesions. Furthermore, since IDH mutations are expected to be homogenous among most or all tumor cells, mIDH expression represents a promising marker for vital tumor cells in IDH-mutated gliomas. As such, mIDH-selective PET tracers could also be used for applications such as tumor delineation during therapy planning, as well as for monitoring the efficiency of novel treatment approaches, detecting recurrence during follow-up examinations, or differentiating treatment-related changes from tumor progression, all of which are of high relevance in clinical practice [41]. In addition, mIDH-selective PET tracers would enable longitudinal in vivo assessment of mIDH expression in preclinical and clinical settings, facilitating further studies on the precise role of IDH mutations for gliomagenesis. Such tools are particularly important because factors such as the tumor microenvironment may have a significant impact on the function and pathophysiological implications of IDH mutations [43]. Finally, mIDH-selective PET tracers could potentially also be used to confirm in vivo target engagement by mIDH-specific drugs, which has proven difficult in glioma patients. Thus, while effective suppression of IDH mutations in peripheral tumors or hematological malignancies can typically be confirmed based on a decrease in circulating 2-HG levels, plasma 2-HG levels in glioma patients are not elevated above the normal concentration [44]. Accordingly, measurement of tumoral 2-HG production would require invasive tissue sampling. Most mIDH-selective inhibitors known to date have been shown or are thought to bind to a common induced-fit pocket (for details, see Section 6 and Section 7). Therefore, radioligands targeting this allosteric pocket should compete for binding with most or all of the candidates currently evaluated in clinical trials and could thus be used for target engagement or occupancy studies. This, in turn, could help to establish whether the lack of clinical responses reported in some cases is due to insufficient target engagement or other factors, such as the narrow time window for therapeutic efficiency of mIDH inhibitors observed in preclinical studies [13].

## 6. Considerations for the Development of mIDH-Selective PET-Tracers

To date, numerous mIDH-selective inhibitors have been developed, and some of them have been shown to effectively reduce 2-HG production in preclinical tumor models and/or in patients [4,46,47]. As will be described in more detail in Section 7, almost all of them effectively inhibit the predominant IDH1_R132H_ mutation and many classes also target additional mutations, which could be advantageous for imaging in glioma patients with non-canonical mutations. However, the suitability of existing inhibitors as leads for tracer development depends on a number of factors, which can be broadly classified into factors related to the general requirements for PET neurotracers and factors related to mIDH as a specific imaging target. In the following two subsections, we will briefly summarize the most important aspects, with a focus on considerations specific to the development of mIDH-selective PET tracers from existing inhibitors. For more detailed discussions of the general requirements for PET neurotracers, readers are referred to several previous reviews on the topic [48,49,50,51].

### 6.1. General Considerations for Development of PET Neurotracers

The development of novel PET tracers for brain imaging is associated with several potential pitfalls and challenges, most of which are related to the strict requirements that need to be fulfilled [48,49,50,51]. For example, a common reason for the failure of PET neurotracers is limited brain entry due to low passive permeability and/or active export across the blood–brain barrier (BBB). In gliomas, the BBB is often partly disrupted, which results in increased permeability to hydrophilic contrast agents and enables tumor visualization by contrast-enhanced MRI [52,53]. However, BBB disruption shows significant intra- and inter-tumoral heterogeneity, and increased uptake of hydrophilic contrast agents is not necessarily predictive of the accumulation of lipophilic drugs or PET tracers [52,53]. In contrast, a number of physicochemical properties (e.g., molecular weight, hydrophobicity, topological polar surface area, basicity, number of hydrogen bond donor atoms) have been shown to be useful for predicting their passive penetration across the intact BBB, especially when combined into a single weighted score (e.g., CNS MPO or CNS PET MPO score) [54,55,56]. In addition, in vitro models of the BBB can be used to obtain a first estimate of the BBB penetration of candidate tracers, although extrapolation of the results to the in vivo situation can be complicated by active transport processes or other factors [57]. Accordingly, it may be sensible to prioritize leads that have already been shown to effectively reduce 2-HG production in brain tumors over compounds that have only been evaluated in hematologic malignancies or peripheral tumors. Even compounds from the former group may not necessarily be optimal candidates for tracer development since there are significant differences between the requirements for PET neurotracers and CNS-targeted therapeutics. For instance, a long plasma half-life and high non-specific binding to brain tissue maintain target-engagement after a single administration and are positive selection traits during drug development but reduce the target-to-background ratio during PET imaging and are undesirable pharmacokinetic (PK) properties for imaging probes [48,57]. In addition, while the pharmacological effects of metabolites are often negligible, the formation of brain-penetrating radiometabolites or in vivo radiodefluorination can degrade imaging quality and interfere with the interpretation of PET studies [49,58,59]. Similarly, slow kinetics of brain penetration may not affect the therapeutic efficiency of drugs dosed continuously but can complicate or prevent PET imaging with short-lived radionuclides such as fluorine-18 [49,57]. Collectively, these factors can significantly hamper tracer development based on existing therapeutics, especially given that some of them are difficult to predict based on the available PK data and/or may be altered by structural modification. Conversely, while factors such as low solubility or poor oral bioavailability increase the efficacious dose of therapeutics and are negative selection traits during drug development, they are not critical for the development of radiotracers, which are typically used in trace amounts and administered intravenously. Likewise, because radiotracers are administered at doses that typically do not cause pharmacological or toxic effects, compounds with dose-limiting toxicity at higher concentrations may still be viable candidates for the development of mIDH-selective PET tracers. Another implication of the low mass doses used for PET imaging is that the affinity and selectivity of candidate radiotracers for their target must be particularly high (e.g., K_d_ values in the low nanomolar range and >30–100-fold selectivity). Furthermore, there should preferably be no significant competition with endogenous ligands (e.g., substrates, co-factors or catalytic metal ions).

### 6.2. Specific Considerations for Tracer Development from Existing mIDH Inhibitors

**Figure 5:**
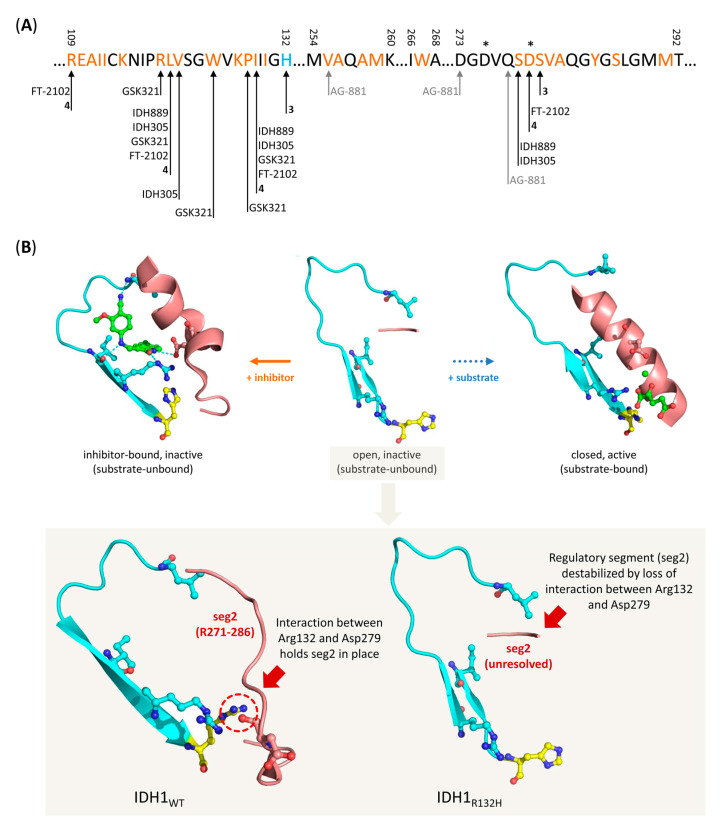
Overview of residues involved in binding of mIDH-selective inhibitors and proposed mechanism for selective inhibition of IDH1_R132H_. (**A**) Amino acid sequence of the allosteric pocket near the substrate binding site of IDH1_R132H_ targeted by most mIDH1-selective inhibitors, and overview of residues involved in inhibitor binding. The mutated histidine residue and residues forming the allosteric pocket are highlighted in blue and orange, respectively, while residues that directly interact with different inhibitors or catalytic Mg^2+^ ions are indicated by arrows or stars, respectively. Note that the pan-mIDH1/2 inhibitor AG-881 binds to an alternative allosteric pocket at the dimer interface but directly interacts with one of the residues (Val_255_) lining the allosteric pocket targeted by mIDH1-selective inhibitors. (**B**) Cartoon representation of the allosteric pocket (indicated in turquoise) and regulatory segment 2 (indicated in red) in IDH1_R132H_ as observed in the crystal structure of the open, inactive (middle, PDB: 3MAR), closed, active (right, PDB: 3INM) or an inhibitor-bound, inactive (left, PDB: 6o2y) conformation. The inhibitor (compound **4**, for details, see Section 7.4) is shown in green, while the mutated residue (His_132_) is shown in yellow. Note that the regulatory segment is destabilized and (due to conformational motions) unresolved in the crystal structure of the inactive conformation (middle), so that inhibitor-binding to the allosteric pocket can lock the enzyme in a quasi-open, inactive conformation (left), while it assumes a long α-helix structure that prevents access to the allosteric pocket in the active conformation (right). Inset: Comparison of the allosteric pocket and regulatory segment 2 in the inactive conformations of IDH1_WT_ (left, PDB: 1T09) and IDH1_R132H_ (right, PDB: 3MAR). Note that interaction between Arg_132_ and Asp_279_ in the wildtype enzyme restricts the conformational flexibility of regulatory segment 2, which may limit access to the allosteric pocket in inactive wildtype enzymes.

With respect to the affinity and selectivity of mIDH-targeted drugs, an important caveat for tracer development is that they have almost exclusively been characterized using IC_50_ values determined by functional assays (e.g., inhibition of wildtype or mutant activity in biochemical or cell-based models) rather than by direct measurement of ligand binding. This is particularly problematic because all known inhibitors with low nanomolar potencies appear to act by allosteric mechanisms, which makes reliable prediction of binding affinities based on IC_50_ values unfeasible. Indeed, target engagement by several inhibitors as measured by cellular thermal shift assays (CETSA) in a glioma cell line transfected with IDH1_R132H_ has been shown to correlate poorly with inhibition in enzymatic assays (R^2^ = 0.18), although a stronger correlation (R^2^ = 0.54–0.78) was observed for inhibition in cellular assays with the same cell line [60]. Since previous studies with radiolabeled inhibitors have generally found a good agreement between IC_50_ values for suppression and K_d_ values for binding to the mutated enzymes in cells as well [61,62], the potency determined in cellular assays may still be a useful surrogate for the affinity of inhibitors for the mutated enzymes. However, prediction of their affinity for the wildtype enzymes based on existing data is more difficult because the selectivity for inhibition of mutant over wildtype enzymes has exclusively been determined in enzymatic assays. Moreover, since inhibition usually does not involve direct interaction with the mutated residues (Figure 5A), the exact mechanisms underlying selectivity for mutated over wildtype enzymes are still incompletely understood and may involve factors apart from selective binding. Indeed, given that the residues involved in binding are typically also present in the wildtype enzyme, it seems conceivable that inhibitors could also bind (with similar affinity) to wildtype enzymes but more effectively inhibit the neomorphic activity of mutant enzymes. Unfortunately, none of the aforementioned studies with radiolabeled inhibitors determined K_d_ values for binding to the wildtype enzymes, and the results of cellular uptake studies to assess mutant selectivity are, in many cases, somewhat inconclusive (see below and Section 7). As such, firm evidence for selective binding to mutated over wildtype enzymes is generally still lacking. However, as summarized in Figure 5 and described in more detail in Section 7, most mIDH1-selective inhibitors have been shown or are thought to target a common induced-fit pocket adjacent to the substrate binding site, which may also be present in the wildtype enzyme but appears to be more accessible in mutated isoforms. While the exact residues involved (Figure 5A), as well as the mode of binding to this pocket (e.g., competitive or non-competitive with respect to α-KG and/or Mg^2+^) can differ between inhibitors, they appear to invariably lock the enzyme in inactive states, as exemplified in Figure 5B [63,64,65,66]. Selectivity for mutant over wildtype enzymes may at least in part arise from mutation-induced destabilization of a regulatory segment (regulatory segment 2 comprised residues 271–286), which is normally held in place by the interaction of Asp_279_ with Arg_132_ (i.e., the mutated residue) and restricts access to the allosteric pocket in inactive wildtype enzymes [66,67,68] (Figure 5B inset). Interestingly, the analogous segment is not similarly destabilized in mutations of IDH2, which may account for the strong mIDH1 preference of most inhibitors [66]. Reduced Mg^2+^ affinity of mutant (K_M_ ≈ 4–10 mm for IDH1_R132H_) compared to the wildtype enzyme (K_M_ ≈ 30 µm) has also been shown to play a role for the selectivity of several mIDH1-selective inhibitors, possibly by reducing competitive binding of Mg^2+^ [64,65] and/or by disrupting Mg^2+^-induced stabilization of the regulatory segment [66,68]. Regardless of the exact mechanisms, these findings collectively suggest that most mIDH1-selective inhibitors indeed bind preferentially to mutated enzymes and could potentially be used for the development of mIDH-selective PET tracers. Nevertheless, given that conclusive experimental data regarding (the degree of) selective binding to mutant over wildtype enzymes is, in most cases, still lacking, it may be advisable to perform initial cellular uptake studies to firmly establish the selectivity of radiolabeled tracer candidates before they are subjected to more detailed in vivo evaluations. In this regard, it is also important to keep in mind that not all systems that have traditionally been used for the development of mIDH-selective drugs may be equally useful for quantifying the selectivity of PET tracers. For example, tumor cell lines engineered to overexpress a mutated IDH isoform may be well suited for assessing how potently an inhibitor suppresses cellular 2-HG production but could be less suitable for certain cellular uptake studies with radioligands. Thus, while most radioligands prepared from mIDH-selective inhibitors showed significantly higher uptake into mIDH-transfected cell lines compared to the corresponding non-transfected cell lines, much less pronounced differences were typically observed when comparing uptake into cell lines carrying a native IDH mutation and the corresponding mIDH-knockout cell lines [61,62,69] (for details, see Section 7). Taken together, these findings suggest that at least some of the apparently selective cellular uptake observed in the transfected cell lines may reflect factors that would not contribute to tumor accumulation in glioma patients, such as a higher overall enzyme concentration in transfected compared to non-transfected cells rather than selective binding to mutant enzymes. The latter could be circumvented by comparing the K_d_ values for mutant and wildtype enzymes determined in saturation binding assays with transfected and non-transfected cell lines, which should be less susceptible to differences in enzyme concentration. However, subsequent in vivo studies in tumor xenograft models should preferably be performed or validated with patient-derived cell lines harboring an endogenous mutation. The application of cells overexpressing the mutated enzymes could lead to an overestimation of the achievable tumor-to-background ratios.

## 7. mIDH-Selective Inhibitors as Potential Leads for PET-Tracer Development

In the remainder of this review, we will provide an overview of the different chemical classes that have been shown to selectively inhibit mutated IDHs with IC_50_ values in the low nanomolar range and discuss their suitability as lead structures for the development of brain-penetrating PET tracers. To this end, we will briefly review the available data on their biochemical, cellular and pharmacokinetic (PK) properties, summarize what is known about their binding mode and, where possible, describe the results from studies with existing radiolabeled analogs. As a starting point for tracer development, we will also provide an overview of the SAR for the most promising inhibitor classes and consider possible strategies for radiolabeling, with a special focus on ^18^F-fluorination. A summary of the inhibitory and PK properties for the most well-characterized representatives of the different chemical classes is provided in Table 1.

### 7.1. Phenylglycine-Derived mIDH1-Inhibitors

**Figure 6:**
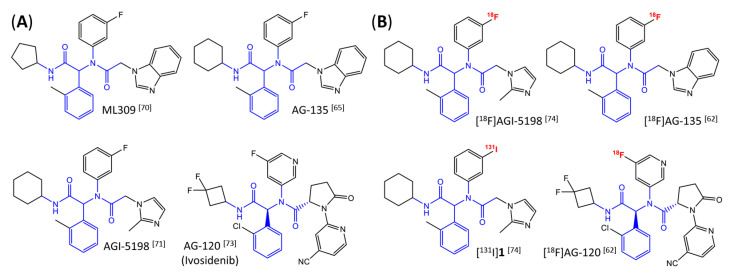
Structure of phenylglycine-based inhibitors and radiolabeled analogs. (**A**) Structure of preclinical and clinical mIDH1-selective inhibitors with an *N*-acetyl phenylglycine amide backbone (highlighted in blue) [65,70,71,73]. (**B**) Overview of radiolabeled phenylglycine-based inhibitors of mIDH1 (radiolabels highlighted in red) [62,74].

Several mIDH1-inhibitors with an *N*-acetyl phenylglycine amide backbone (Figure 6A) developed by Agios Pharmaceuticals have been shown to potently inhibit IDH1_R132H_ (and in some cases other mutations in codon 132), with excellent selectivity over the wildtype enzyme (Table 1) [70,71,72,73]. In addition to preclinical compounds such as ML309, AGI-5198 and AG-135, they include AG-120 (Ivosidenib), which has been FDA-approved for the treatment of acute myeloid leukemia (AML) and is in clinical trials for the treatment of various solid tumors, including gliomas [46]. Although high-resolution crystallographic data showing the exact binding mode of phenylglycine-based inhibitors is still lacking, cryo-EM data, computational predictions and functional data suggest that they bind to the allosteric pocket adjacent to the substrate binding site, which may disrupt the interaction of mutant enzymes with catalytic Mg^2+^ ions [64,89,90]. In addition to promising biochemical and cellular inhibitory potencies, the phenylglycine-based inhibitors generally contain a fluorophenyl or fluoropyridine motif so that radiofluorinated compounds can be prepared without structural modification (Figure 6A). Unfortunately, the preclinical compounds in this group also have several unfavorable PK properties for PET imaging, such as rapid metabolic clearance, a high degree of plasma protein binding and lack of brain penetration (Table 1), suggesting that they may not be optimal candidates for tracer development. Consistent with this assumption, the ^18^F-labeled isotopologs of AGI-5198 and AG-135 ([^18^F]AGI-5198 and [^18^F]AG-135, Figure 6B) have been found to show selective uptake into IDH1_R132H_- or IDH1_R132C_-transfected tumor cells in vitro but no brain entry and insufficient tumor uptake and retention in mice bearing subcutaneous IDH1_R132H_ or IDH1_R132C_ tumor xenografts [62,74]. Furthermore, even though cellular uptake of [^18^F]AGI-5198 was mIDH1-selective when comparing IDH1_R132H_-transfected and non-transfected tumor cells, almost no difference in uptake was observed between patient-derived tumor cells bearing a native IDH1_R132H_ mutation and the corresponding IDH1_R132H_ knockout cells [74], which could indicate high non-specific binding and/or significant binding to the wildtype enzyme. Interestingly, the replacement of the fluorine atom in AGI-5198 with radioiodine resulted in selective uptake into both the transfected and patient-derived IDH1_R132H_ mutated tumor cells in vitro, while in vivo uptake of the resulting radioligand ([^131^I]1, Figure 6B) into IDH1_R132H_-transfected and non-transfected tumor xenografts was similar [74]. AG-120, which exhibits significantly improved metabolic stability and a reduced degree of plasma protein binding (Table 1), could be a more promising starting point for tracer development. AG-120 has been shown to potently inhibit a broad spectrum of IDH1 mutations (IC_50_ < 20 nm for R132H, R132C, R132G, R132L and R132S) in a manner that is non-competitive with respect to the substrate α-KG [60,73], whereas inhibition by other phenylglycine-based inhibitors, such as ML309 or AGI-5198, is substrate-competitive [60,70,71]. In mice bearing subcutaneous IDH1_R132C_ or IDH1_WT_ tumors, ^18^F-labeled AG-120 ([^18^F]AG-120, Figure 6B) showed selective accumulation in the IDH mutated tumors [62], suggesting that it may be useful for non-invasive assessment of the IDH1 mutation status in peripheral tumors. However, a potential drawback for glioma imaging is that AG-120 is an avid substrate for active export in P-gp-transfected MDCK cells [60] and shows low in vivo brain exposure in healthy rats (Table 1) [73], as well as in the aforementioned subcutaneous mouse tumor model [62]. Since brain exposure was only examined in animals without IDH-mutated brain tumors and the exact kinetics of brain penetration were not reported, the latter findings could potentially reflect a relatively rapid clearance from normal brain tissue (which would be advantageous for PET imaging if tumor accumulation is sufficiently rapid as well). Moreover, it has been proposed that brain penetration of AG-120 is increased in glioma patients with partial BBB disruption [73], and preliminary results from clinical trials reported on various meetings indicate that it may be effective against certain low-grade gliomas and is able to suppress tumoral 2-HG production in patients and orthotopic brain tumor models [91,92,93,94,95]. On the other hand, considering the long plasma half-life of up to 19 h observed in preclinical PK studies (Table 1) and the fact that therapeutic treatment involves continuous dosing, these findings may also reflect a slow tumoral accumulation of AG-120 over time, which could complicate or prevent PET imaging using probes labeled with short-lived radionuclides. In addition, the upregulation of P-gp is a known mechanism of drug resistance in cancer that may reduce tumor accumulation of [^18^F]AG-120 and related tracers in patients with acquired resistance [96]. As such, while further studies are needed to firmly establish the extent and time-course of accumulation in IDH1 mutated brain tumors, the available data suggest that [^18^F]AG120 may not be an optimal candidate for glioma imaging in patients.

### 7.2. Pyrimidinyl-Oxazolidinone-Based mIDH1-Inhibitors

**Figure 7:**
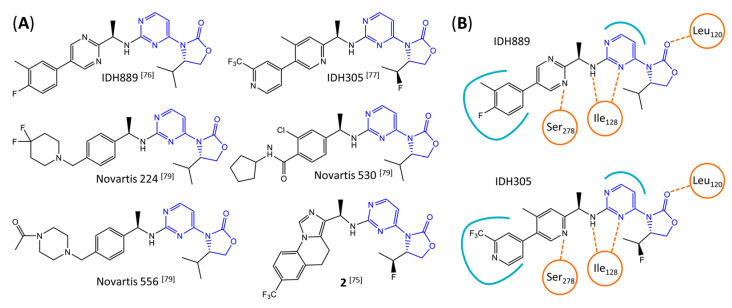
Structure of pyrimidinyl-oxazolidinone-based inhibitors and their interaction with IDH1_R132H_. (**A**) Structure of preclinical and clinical mIDH1-selective inhibitors with a pyrimidinyl-oxazolidinone backbone (indicated in blue) [75,76,77,79]. (**B**) Scheme illustrating inhibitor-protein interactions in the crystal structures of IDH889 (top, PDB: 5TQH) or IDH305 (bottom, PDB: 6B0Z) in complex with IDH1_R132H_. Amino acid residues that directly interact with the inhibitors are shown in orange circles, with dotted lines indicating the formation of hydrogen bonds. In addition, key hydrophobic interactions of the inhibitors with the protein are indicated in turquoise.

Novartis has pursued potent mIDH1-selective inhibitors based on a 3-pyrimidin-4-yl-oxazolidin-2-one scaffold (Figure 7A). The initial lead compound IDH889 showed good in vitro inhibitory potency and selectivity but unfavorable PK properties, such as high plasma protein binding and rapid metabolic clearance (Table 1) [76]. Further optimization led to the identification of IDH305, a brain-penetrant mIDH1-selective inhibitor that effectively reduced 2-HG production and suppressed tumor-growth in subcutaneous IDH1_R132H_ and IDH1_R132C_ xenograft models [77]. Crystallographic data of IDH889 or IDH305 in complex with IDH_R132H_ shows that both inhibitors bind to the allosteric pocket adjacent to the active site. The aminopyridine moieties and the carbonyls of the oxazolidinone motif form hydrogen bonds with Ile_128_ and Leu_120_, respectively, while nitrogen atoms in the pyrimidine (IDH889) or pyridine (IDH305) ring form a hydrogen bond with the hydroxyl group of Ser_278_ in the regulatory segment (Figure 7B) [76,77]. A phase I clinical trial with IDH305 for the treatment of various advanced malignancies harboring IDH_R132_ mutations was prematurely terminated in 2016 due to the occurrence of reversible hepatotoxicity, which was manageable with dose modification but predicted to result in a narrow therapeutic window [78]. Since dose-limiting toxicity should not be an issue for use as a PET tracer, IDH305 may still be a promising starting point for tracer development, especially considering that it could be radiofluorinated without structural modification and has several favorable PK properties for PET imaging (e.g., high metabolic stability, low plasma protein binding and good brain penetration) (Table 1). However, a relatively low fraction of unbound drug (f_u,brain_) was observed in in vitro assays with brain tissue homogenate, suggesting that it may exhibit high non-specific binding (Table 1). Apart from IDH305, a number of other mIDH1-inhibitors with the same backbone structure, including several compounds containing a fluorine substituent (Table 2), have been described, although only some of them have been characterized in more detail (Table 1) [60,75]. While several of the analogs disclosed by Novartis (Figure 7A) have been shown to potently and selectively inhibit 2-HG production by IDH1_R132H_ and IDH1_R132C_, they exhibited limited metabolic stability in microsomal assays, and in vivo metabolism or brain penetration have not been studied (Table 1). More suitable for the development of PET tracers for glioma imaging could be some of the IDH305-derived inhibitors recently described by Cao et al. that were specifically developed with the aim of identifying brain-penetrating drugs for glioma treatment [75]. One of the inhibitors from this series (compound **2** in Figure 7A) was evaluated in a subcutaneous IDH1_R132C_ mouse xenograft model and showed significantly higher tumor accumulation as well as brain exposure than the phenylglycine-based inhibitor AG-120 [75]. In addition, compound **2** exhibited low in vivo hepatic metabolism yet a relatively fast clearance from circulation (Table 1), suggesting that this inhibitor or its analogs may be promising starting points for tracer development. Apart from replacing the aliphatic or aromatic fluorine substituents present in many pyrimidinyl-oxazolidinone-based inhibitors with fluorine-18, some other radiolabeling strategies could be envisioned based on the existing SAR and crystallographic data. For example, radiofluorination could be performed in positions 5 or 6 of the pyrimidine ring, which are oriented toward a hydrophobic region of the allosteric pocket (Figure 7B) and have been shown to tolerate halogenation without changes in the biochemical IC_50_ values (Table 2). Similarly, substituents attached to the benzylic amine linker are located in a hydrophobic sub-pocket that can accommodate various (substituted) aryl or heteroaryl motifs, suggesting that these parts of the inhibitors could potentially be modified by the introduction of a small ^18^F-labeled alkyl group or be replaced by a suitable ^18^F-labeled aryl or heteroaryl scaffold. However, to date, no radiolabeled pyrimidinyl-oxazolidinone-based inhibitors have been described, and their suitability as imaging agents remains to be evaluated.

### 7.3. Aminobenzimidazole-Derived mIDH1-Inhibitors

**Figure 8:**
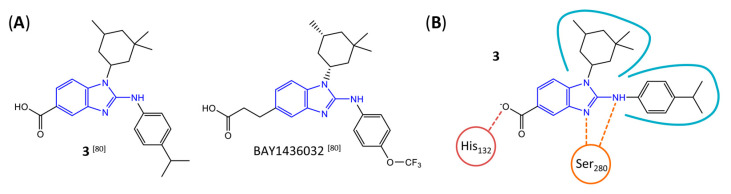
Structure of aminobenzimidazole-based mIDH1 inhibitors and their interaction with IDH1_R132H_. (**A**) Structure of BAY1436032 and a structural analog from the lead optimization program (**3**) with the same 2-aminobenzimidazole backbone (indicated in blue) [80]. (**B**) Scheme illustrating inhibitor-protein interactions in the crystal structure of **3** in complex with IDH1_R132H_ (PDB: 5LGE). Amino acid residues that directly interact with the inhibitor are shown in red or orange circles, with dotted lines indicating the formation of hydrogen bonds (orange) or salt bridges (red), respectively. In addition, key hydrophobic interactions of the inhibitor with the protein are indicated in turquoise.

Based on an initial screening of over 3 million compounds and subsequent optimization of the most promising 2-aminobenzimidazol-based lead structure, Bayer developed BAY1436032 (Figure 8A), which is a potent pan-inhibitor of codon 132-mutated IDH1 enzymes [80]. Thus, in HEK293 cells expressing different mutations, BAY1436032 (500 nm) inhibited 2-HG production by various IDH1 mutations (R132H, R132C, R132G, R132S and R132L) with equal efficiency (~60–70%) but produced no or marginal (<20%) inhibition of different IDH2 mutations (R172K, R172W, R172M) [80]. BAY1436032 has also been shown to potently inhibit 2-HG production by several IDH1 mutations in engineered or patient-derived tumor cell lines, with IC_50_ values in the low nanomolar range (Table 1). No crystal structure for BAY1436032 bound to any of the IDH1 mutations has been reported to date. However, co-crystals of a structural analog from the lead optimization program (compound **3** in Figure 8A) in complex with IDH1_R132H_ indicate that it also targets the allosteric pocket adjacent to the active site, where the 2-aminobenzimidazole scaffold could form hydrogen bonds with Ser_280_ in the regulatory segment, while the carboxyl group could build a salt bridge with His_132_ (Figure 8B). Thus, aminobenzimidazoles such as BAY1436032 may be unique among the allosteric inhibitors known to date owing to their direct interaction with the mutated residues, which is particularly interesting given the nearly equal potency against many different IDH1 mutations. However, interaction with His_132_ may not be critical for high-affinity binding, as several potent analogs lacking a carboxyl group have been described in the patent literature (Table 3) [98]. Due to its favorable PK properties, BAY1436032 has also been evaluated in clinical trials for the treatment of AML and various IDH1 mutated solid tumors, including gliomas, and showed a favorable safety profile but low overall response rates [44,81]. Nevertheless, as mentioned in Section 2 and discussed in more detail elsewhere [13,14], the therapeutic efficiency of mIDH-selective drugs as anti-cancer agents remains controversial and may be limited to early stages of tumorigenesis, so that a lack of clinical responses does not necessarily indicate insufficient target engagement. Consistent with this notion, BAY1436032 induced a rapid and robust decrease in plasma 2-HG levels in most patients with peripheral tumors, suggesting that limited clinical effects in these patients were not related to insufficient target engagement [44]. In addition, the best therapeutic efficiency was observed in patients with low-grade gliomas, although the effects on tumoral 2-HG production in this subgroup could not be assessed [44]. BAY1436032 has also been shown to effectively penetrate the BBB in healthy rats, and to significantly reduce tumoral 2-HG production and increase survival in mice bearing intracerebral IDH1_R132H_ glioma xenografts, providing further evidence for effective target engagement in the brain [80]. In addition, the compound exhibits low hepatic metabolism yet a relatively fast clearance from circulation (Table 1), suggesting that it may be a viable starting point for the development of mIDH1-selective PET tracers that could detect a wide range of mutations in codon 132. A potential disadvantage for PET imaging is that inhibition in biochemical assays was competitive with respect to α-KG (~10-fold change in IC_50_ value for increasing α-KG concentrations from 0.25 to 10 mm) [80]. This may reflect the fact that substrate-binding induces a loop-to-helix transition of the regulatory segment that occludes access to the allosteric pocket (see Figure 5B). However, this does not necessarily preclude the use of radiolabeled BAY1436032 analogs for glioma imaging, because the concentration of α-KG in IDH-mutated gliomas is expected to be low (<0.1 μmol/g) [5]. Radiolabeling could be achieved without structural modification by incorporating fluorine-18 into the trifluoromethoxy moiety of BAY1436032 (see, e.g., [99,100]). Potential drawbacks of this approach are the low molar activities often associated with CF_3_-labeling methods and a relatively high risk of in vivo defluorination due to the metabolic instability of the CF_3_ group. Alternative radiolabeling strategies could be developed based on the available SAR and crystallographic data. While a complete list of the numerous analogs described in the patent literature is beyond the scope of this article, Table 3 provides an overview of some of the more potent (i.e., IC_50_ ≤ 100 nm) compounds to illustrate general trends in the SAR. It can be seen that the introduction of fluorine or other small substituents at positions 4 or 6 of the benzimidazole scaffold was usually not associated with adverse effects on the potency, suggesting that a small radiolabel such as fluorine-18 or an ^18^F-labeled alkyl group could be introduced at these positions. Similarly, the trifluoromethoxyphenyl group of BAY1436032, which is expected to be located in a hydrophobic sub-pocket of the enzyme (Figure 8B), could potentially be modified by substitution of the CF_3_ group with an ^18^F-labeled alkyl or an [^18^F]fluorosulfonate group. Alternatively, the entire 4-trifluoromethoxyphenyl substituent or the 3,3,5-trimethyl-cyclohexyl group (which is also located in a hydrophobic sub-pocket, Figure 8B) could be replaced by a suitable ^18^F-labeled cycloalkyl, aryl or heteroaryl scaffold, although the effects of these modifications on the inhibitory potency are more difficult to predict. Finally, it is worth noting that the presence of a carboxyl group often reduces the rate (or extent) of brain penetration [101,102], which could impede PET imaging with short-lived radionuclides. BAY1436032 analogs lacking a (free) carboxyl group have in several cases been shown to retain high inhibitory potency (Table 3). Such compounds may therefore be particularly promising leads for the development of aminobenzimidazole-based PET tracers for glioma imaging.

### 7.4. Quinolinone-Based mIDH1-Inhibitors

**Figure 9:**
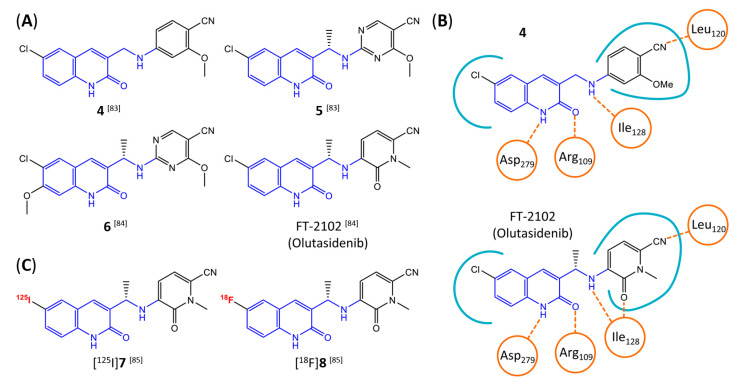
Structure of quinolinone-based inhibitors and their interaction with IDH1_R132H_. (**A**) Structure of preclinical and clinical mIDH1-selective inhibitors with a 1H-quinolin-2-one backbone (indicated in blue) [83,84]. (**B**) Scheme illustrating inhibitor-protein interactions in the crystal structures of compound **4** (top, PDB: 6O2Y) and FT-2102 (bottom, PDB: 6U4J) in complex with IDH1_R132H_. Amino acid residues that directly interact with the inhibitors are shown in orange circles, with dotted lines indicating the formation of hydrogen bonds. In addition, key hydrophobic interactions of the inhibitors with the protein are indicated in turquoise. (**C**) Structure of radiolabeled mIDH inhibitors, with the radiolabels indicated in red [85].

In a screen of over 400,000 compounds, Forma Therapeutics identified the 1*H*-quinolin-2-one scaffold as a promising backbone for the development of mIDH1-selective inhibitors [83]. Subsequent SAR exploration led to the discovery of an early lead compound (compound **4** in Figure 9A) that inhibits IDH1_R132H_ with moderately high potency and excellent (>700-fold) selectivity over the wildtype enzyme but exhibits relatively low microsomal stability (Table 1) [83]. The crystal structure of compound **4** in complex with IDH1_R132H_ demonstrated that it also binds to the allosteric pocket near the active site, where the quinolinone core forms hydrogen bonds with Arg_109_ and Asp_279_, the benzylic amine linker forms a hydrogen bond with Ile_128_ and the cyano group forms a hydrogen bond with Leu_120_ [83] (Figure 9B). SAR studies with this early lead structure revealed that the addition of an (*S*)-methyl group at the α-position of the benzylic amine linker between the quinolinone core and the right-hand side (RHS) cyanobenzene moiety significantly improves the potency (Table 4) and reduces microsomal metabolism, presumably by constraining the linker to adopt the bioactive conformation while blocking metabolism at the α-position of the benzylic group [83]. Additional replacement of the RHS portion with various phenyl, pyridine or pyrimidine rings containing a *p*-CN and *m*-MeO or -Me group invariably resulted in compounds with very high biochemical potencies against IDH1_R132H_ [83]. Likewise, substitution in the 7-position of the quinolinone core (Table 4) was well tolerated and typically increased the potency against IDH1_R132H_ in the case of large substituents such as cyclopropyl, methoxy or cyclopropylmethoxy groups, albeit in some cases at the expense of reduced solubility and/or metabolic stability [83,84]. Taken together, these findings led to the development of the preclinical compounds **5** and **6** as well as the clinical candidate FT-2102 (Olutasidenib) (Figure 9A), which is currently in clinical trials for the treatment of AML and various solid tumors, including gliomas. All three compounds have been shown to potently inhibit 2-HG production by several IDH1 mutations (R132H, R132C, R132G, R132L) in biochemical and cellular assays, with IC_50_ values in the low nanomolar range and excellent selectivity (>196-fold) over the wildtype enzyme (Table 1) [83,84]. In addition, they exhibit favorable PK properties, such as high in vivo stability, a high passive permeability in artificial membrane assays, lack of significant efflux in P-gp-transfected MDCK cells and (at least for FT-2102) a relatively high brain-to-plasma ratio in preclinical studies (Table 1) [83,84]. The crystal structure of FT-2102 in complex with IDH1_R132H_ shows that it directly interacts with the same residues in the allosteric pocket as the early lead compound **4**, except that the carbonyl oxygen in the RHS pyridinone ring forms an additional hydrogen bond with Ile_128_, which may contribute to the improved potency [84] (Figure 9B). A small multi-center clinical trial in patients with relapsed or refractory IDH1 mutated gliomas observed no dose-limiting toxicity and provided preliminary evidence for effective brain penetration and therapeutic efficacy of FT-2102 [103], although these results need to be confirmed by further studies. Nevertheless, the quinolinone-based inhibitors are a promising chemical class for tracer development since they could be used to detect a wide range of IDH1 mutations present in gliomas and generally exhibit favorable PK properties. In addition, even though none of the promising preclinical and clinical candidates described above contains a native fluorine atom, the class is well characterized in terms of its binding mode (Figure 9B) and SAR (Table 4), which should facilitate the development of radiolabeling strategies. For example, positions 6 and 7 of the quinolinone core are oriented towards a partially exposed, hydrophobic region of the allosteric pocket and can accommodate relatively large substituents, making them promising positions for radiofluorination. Indeed, during lead optimization, neither replacement of the 6-chloro substituent in compound **4** by fluorine, nor introduction of an additional 7-fluoro substituent in compound **5** or FT-2102 had major adverse effects on the biochemical or cellular potencies against IDH1_R132H_ and IDH1_R132C_ (≤2-fold increase in IC_50_ values, Table 4), and the respective analogs were mainly omitted due to low solubility [83,84]. Accordingly, the first and so far only radiolabeled quinolinone-based inhibitors were developed by replacing the 6-chloro substituent in FT-2102 with fluorine-18 or iodine-125 [85]. In the same work, the potency of the corresponding non-labeled compounds was studied. In line with the above findings, the structural modification resulted in no change in the potency for the iodinated analog and only a modest increase in the biochemical IC_50_ values (from 5 to 23 nM for IDH1_R132H_ or 178 nM to 579 nM for IDH1_R132C_) for the fluorinated analog [85]. The authors also evaluated the corresponding (*R*)-methyl enantiomers, which inhibited IDH1_R132H_ with at least 30-fold lower potency (Table 4), consistent with the suggested role of the (*S*)-methyl substituted benzylic linker for the bioactive inhibitor conformation described above. All four analogs had no appreciable effect on the corresponding wildtype enzymes, with IC_50_ values of >10 µM for both IDH1 and IDH2. In addition, the radiolabeled isotopologs of the more potent (*S*)-methyl enantiomers (Figure 9C) were found to be stable for at least two hours when incubated in saline or buffer solutions at room temperature or in mouse serum at 37 °C [85]. While further biological evaluation of both radiolabeled inhibitors is pending, these preliminary results support the notion that quinolinone-based inhibitors may be a useful starting point for tracer development. As described above, relatively large substituents in position 7 of the quinolinone core often improved the biochemical and cellular IC_50_ values during lead optimization (Table 4). Consequently, radiolabeled analogs with enhanced inhibitory potency could potentially be obtained by introducing ^18^F-labeled alkyl, aryl or heteroaryl scaffolds in this position, although this might, in some cases, negatively affect the metabolic stability and/or brain penetration. The effects of substituents in the adjacent position 8 are less established, but the available data suggest that it should also be possible to introduce a radiolabel in this position without adverse effects on mIDH1 affinity (Table 4). Finally, as an alternative to radiolabeling of the quinolinone core, the RHS portion in FT-2102 or its analogs could potentially be replaced with ^18^F-labeled substituents containing suitably placed cyano and carbonyl groups to maintain the interactions with Leu_120_ and Ile_128_ observed in the crystal structures (Figure 9B).

### 7.5. Tetrahydropyrazolopyridine-Based mIDH1-Inhibitors

**Figure 10:**
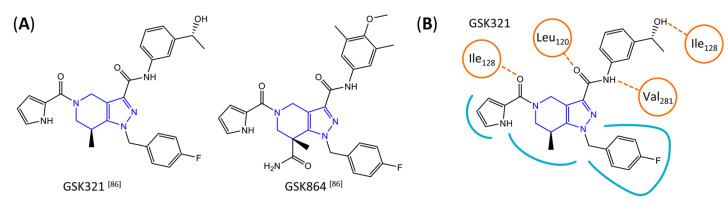
(**A**) Structure of tetrahydropyrazolopyridine-based inhibitors and their interaction with IDH1_R132H_. Structure of preclinical mIDH1-selective inhibitors with a tetrahydropyrazolopyridine backbone (indicated in blue) [86]. (**B**) Scheme illustrating inhibitor-protein interactions in the crystal structure of GSK321 in complex with IDH1_R132H_ (PDB: 5DE1). Amino acid residues that directly interact with the inhibitor are shown in orange circles, with dotted lines indicating the formation of hydrogen bonds. In addition, key hydrophobic interactions of the inhibitor with the protein are indicated in turquoise.

A further class of potent mIDH1-selective inhibitors comprises the tetrahydropyrazolopyridine-based preclinical compounds GSK864 and GSK321 developed by GlaxoSmithKline (Figure 10A). Both compounds have been shown to inhibit 2-HG production by different mIDH1 enzymes (R132H, R132C and R132G) in biochemical assays with low nanomolar IC_50_ values and moderate to high selectivity over the wildtype enzyme (Table 1) [60,86]. Based on the crystal structure of GSK321 bound to IDH1_R132H_, these inhibitors also occupy the allosteric pocket near the active site, where they form hydrogen bonds with Leu_120_ and Ile_128_ as well as with Val_281_ and Gly_284_ in the regulatory segment (Figure 10B). Despite not binding to the same site as the substrate or co-factor, inhibition is competitive with respect to α-KG [60,86] and Mg^2+^ [64], which may be related to occlusion of the allosteric pocket by the regulatory segment in the active conformation, as described above for IDH305. Moreover, while GSK864 has been shown to effectively reduce 2-HG production by IDH1_R132H_ and IDH1_R132C_ in an AML xenograft model, the efficiency against 2-HG production in solid tumors as well as in vivo brain exposure of this compound have not been studied. However, the results of in vitro assays with artificial membranes and Caco-2 cells point to a relatively low passive permeability of this compound. This could explain why the IC_50_ values for suppression of different IDH1 mutations in cells were typically much higher than those observed in biochemical assays (Table 1) and suggests that tumor accumulation and/or BBB-penetration may be low as well. As such, even though both members of this class contain an aromatic fluorine atom and could be radiofluorinated without structural modification, tetrahydropyrazolopyridine-based inhibitors may represent suboptimal candidates for the development of PET tracers for glioma imaging.

### 7.6. Butyl-Phenyl Sulfonamide-Based mIDH1-Inhibitors

**Figure 11:**
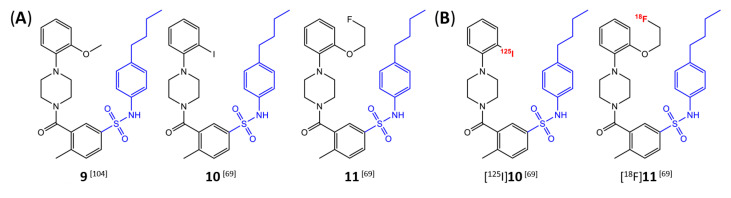
Structure of butyl-phenyl sulfonamide-based inhibitors and radiolabeled analogs. (**A**) Structure of mIDH1-inhibitors with a butyl-phenyl sulfonamide backbone (highlighted in blue) [69,104]. (**B**) Radiolabeled butyl-phenyl sulfonamide-based inhibitors with the radiolabels indicated in red [69].

Butyl-phenyl sulfonamides were one of the first classes of mIDH-selective inhibitors described in the patent literature [104]. Among others, the lead compound **9** (Figure 11A) and several iodinated or fluorinated analogs were prepared and evaluated for their inhibitory potency against IDH1_R132H_ in biochemical assays [69]. Whereas the results showed that substitution of the *o*-MeO group in the lead structure with an iodo or fluoroethoxy group (compounds **10** and **11** in Figure 11A) did not negatively affect inhibition, the IC_50_ values for the parent compound as well as all tested analogs exceeded 1 µm, and effects on the wildtype enzyme were not examined [69]. In addition, although incubation with the *ortho*-iodinated or -fluoroethoxylated derivatives effectively reduced 2-HG production in a patient-derived astrocytoma cell line harboring a native IDH1_R132H_ mutation by approximately 40–50%, no significant difference in cellular uptake compared to the respective IDH1_WT_ cell line was observed for the ^125^I- or ^18^F-labeled compounds (Figure 11B) [69]. Furthermore, cellular uptake into both cell lines was significantly increased by co-incubation with increasing concentrations of the respective non-radioactive reference compounds or by decreasing the concentration of serum in the incubation medium, indicating a high degree of non-specific binding and/or lack of selectivity for mutant over wildtype enzymes [69]. Finally, while biodistribution studies with the ^125^I-labeled candidate showed some favorable tissue distribution characteristics (e.g., rapid clearance from blood and no evidence for in vivo dehalogenation), brain uptake of the radioligand was very low, suggesting a lack of BBB-penetration by this class of inhibitors [69]. Taken together, these findings suggest that butyl-phenyl sulfonamide analogs are unlikely to be suitable as agents for glioma imaging.

### 7.7. Aminotriazine-Based mIDH2- and mIDH1/2-Inhibitors

**Figure 12:**
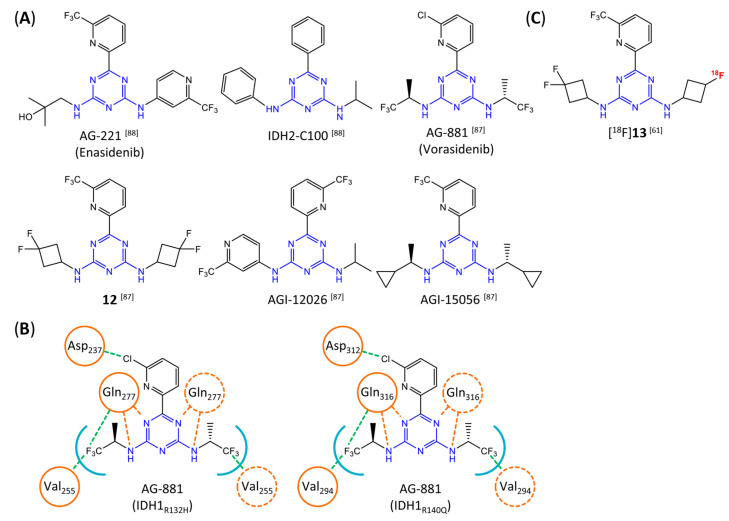
Structure of aminotriazine-based inhibitors and their interaction with IDH1_R132H_ and IDH2_R140Q_. (**A**) Structure of preclinical and clinical mIDH1/2-selective inhibitors with an aminotriazine backbone (indicated in blue) [87,88]. (**B**) Scheme illustrating inhibitor-protein interactions in the crystal structures of AG-881 in complex with IDH1_R132H_ (left, PDB: 6VEI) or IDH2_R140Q_ (right, PDB: 6VFZ) homodimers. Amino acid residues that directly interact with the inhibitors are shown in orange circles, with dotted circles indicating residues belonging to the second monomer and dotted lines indicating the formation of hydrogen (orange) or halogen (green) bonds, respectively. In addition, key hydrophobic interactions of the inhibitors with the protein are indicated in turquoise. (**C**) Structure of the only radiolabeled analog of the inhibitors shown in A that has been described, with the radiolabel indicated in red [61].

In contrast to the compounds described above, aminotriazine-based inhibitors developed by Agios Pharmaceuticals selectively inhibit mIDH2 (AG-221/Enasidenib and preclinical analogs such as IDH2-C100) or act as pan-inhibitors against mIDH1 and mIDH2 (AG-881/Vorasidenib and preclinical analogs such as AGI-12026 or AGI-15056) (Table 1). AG-221 (Enasidenib) (Figure 12A) was developed by optimizing an early lead structure identified in high-throughput screens for inhibitors of the IDH2_R140Q_ mutation and was the first mIDH2-selective inhibitor to receive FDA approval for the treatment of AML [46]. Given the selectivity for IDH2 mutations that are only found in hematologic malignancies or peripheral tumors as well as a low degree of brain penetration, AG-221 and preclinical mIDH2-selective inhibitors such as IDH2-C100 (Figure 12A) are of less interest for the development of PET probes for glioma imaging. However, several compounds evaluated during lead optimization also showed activity against IDH1 mutations. Further structural modification to increase brain penetration and mIDH1 affinity ultimately led to AG-881 (Figure 12A), which is an inhibitor of most mIDH1 and mIDH2 enzymes that have been detected in gliomas (Table 1). The compound has also been shown to penetrate the BBB in several preclinical species and to suppress 2-HG production in an orthotopic glioma mouse model, demonstrating effective target engagement in the brain [87]. As a result, AG-881 is currently being evaluated by clinical trials in glioma patients, with initial results suggesting that it is well tolerated, effectively suppresses tumoral 2-HG production in the brain and may have antitumor activity in certain patient populations [105,106]. Consistent with the activity against mIDH1 and mIDH2, crystal structures of AG-881 bound to IDH1_R132H_ or IDH2_R140Q_ homodimers show that this compound does not bind to the same induced-fit pocket as mIDH1-selective inhibitors but rather to a nearby allosteric pocket that is located at the dimer interface. At this pocket, the aminotriazine core forms two hydrogen bonds with Gln_277_ (IDH1_R132H_) or Gln_316_ (IDH2_R140Q_) from both monomers. In addition, each of the two aliphatic CF_3_ groups forms a halogen bond with Val_255_ (IDH1_R132H_) or Val_294_ (IDH2_R140Q_) from one monomer. One of the CF_3_ groups forms an additional halogen bond with Gln_277_ (IDH1_R132H_) or Gln_316_ (IDH2_R140Q_) from one monomer, and the chloropyridine moiety forms a halogen bond with Asp_273_ (IDH1_R132H_) or Asp_312_ (IDH2_R140Q_) from the same monomer (Figure 12B). Inhibition is thought to result from conformational changes that induce steric hindrance within the substrate binding site and lock the enzymes in an inactive conformation [107]. While the mode of binding of AG-881 with respect to α-KG has not been reported, data on AG-221 (which binds to the same allosteric pocket in mIDH2) and a radiolabeled analog (see below) indicate that aminotriazine-based inhibitors bind non-competitively with respect to the substrate [61,88]. Interestingly, two alternative (symmetric) conformations of AG-881 could be modeled in the co-crystal structure with IDH2_R140Q_ but not IDH1_R132H_, suggesting subtle differences in the pockets. These differences may account for the remarkably different inhibition kinetics of mIDH1 and mIDH2 observed in functional studies. Thus, while the inhibition of mIDH1 by AG-881 in biochemical assays shows rapid-equilibrium characteristics (IC_50_ = 6 or 8 nm after 1 h or 16 h preincubation for IDH1_R132H_), inhibition of mIDH2 is markedly time-dependent (IC_50_ = 118 or 12 nm after 1 h or 16 h preincubation for IDH2_R140Q_) [87]. Inhibition of the respective wildtype enzymes by AG-881 is also time-dependent, with IC_50_ values ranging from 190 nM (1 h preincubation) to 4 nM (16 h preincubation) for IDH1 and 374 nM (1 h preincubation) to 31 nM (16 h preincubation) for IDH2 [87]. An important implication of these findings is that the selectivity of AG-881 for mutated over wildtype IDH1 enzymes may also be time-dependent. Thus, based on the above data from biochemical assays, the selectivity for suppression of 2-HG production by IDH1_R132H_ over IDH1_WT_ homodimers would amount to approximately 32-fold after 1 h but decrease to 0.5-fold after 16 h. The situation is further complicated by the fact that IDH mutations in gliomas are almost always heterozygous, which means that tumor cells are likely to contain both mutant homodimers and mutant/wildtype heterodimers, the exact ratio of which has not been established [4,108]. As expected, based on the fact that both counterparts of a dimer are involved in the binding of AG-881, its effects on 2-HG production by homo- and heterodimers have been shown to differ, with IDH1_R132H/WT_ heterodimers demonstrating lower IC_50_ values than IDH1_R132H_ homodimers whereas IDH2_R140Q/WT_ heterodimers display higher IC_50_ values than IDH2_R140Q_ homodimers (Table 1).

Taken together, these factors make it difficult to extrapolate biochemical and cellular efficiency to the in vivo situation, especially given that most cellular studies have been performed in engineered cell lines overexpressing the mutant enzyme. With respect to the development of PET tracers, they also indicate that the advantage of AG-881 to target both mIDH1 and mIDH2 could at least in part be offset by a relatively low mutant selectivity (~2–3-fold for mIDH2 and ≤32-fold for mIDH1 depending on the time after administration) and complex binding kinetics. However, while the frequency of mIDH2 in gliomas is low, recent studies suggest that isoform switching from mIDH1 to mIDH2 (and vice versa) may be an important mechanism of acquired resistance to mIDH inhibition [109,110]. Increased use of therapeutic strategies targeting mIDH1 may therefore also increase the proportion of glioma patients harboring IDH2 mutations, underscoring the need for the development of brain-penetrating PET tracers that can reliably detect mutations in both isoforms. As such, aminotriazine-based inhibitors remain a promising chemical class for tracer development, especially given that their SAR has been studied in some detail and that several highly potent, fluorine-containing analogs apart from AG-881 have been described (Table 5) [87,88]. [^18^F] **13**, as the first radiofluorinated mIDH inhibitor of this class (Figure 12C), was derived from the preclinical compound **12** (Figure 12A) by replacing one of the difluorocyclobutyl substituents by an ^18^F-labeled monofluorocyclobutyl moiety [61]. Competitive inhibition assays with the resulting radioligand in an IDH1_R132H_-transfected glioma cell line yielded an IC_50_ value of 54 nm for the displacement of the corresponding non-labeled compound, which is somewhat lower than the cellular IC_50_ value of 1 nm for suppression of 2-HG production by the parent compound [61]. In good agreement with this result, saturation binding assays in a patient-derived glioma cell line carrying a native IDH1_R132H_ mutation yielded a K_d_ value of 40 nm. Cellular uptake of the radioligand into the IDH1_R132H_-transfected glioma cell line was non-competitive with respect to a cell-permeable α-KG analog but almost completely blocked by co-incubation with the non-labeled reference compound or AG-221, suggesting that non-specific uptake is low [61]. Interestingly, co-incubation with AGI-5198 or GSK864 blocked cellular uptake as well [61], suggesting that radiolabeled aminotriazines could potentially be used for target engagement studies with mIDH1-selective inhibitors. More importantly, while uptake into the IDH1_R132H_-transfected glioma cell line was 7.8-fold higher than uptake into the corresponding non-transfected cell line, uptake into the patient-derived derived IDH1_R132H_ cell line was only about two-fold higher than uptake into the corresponding IDH1_R132H_ knockout cell line [61]. In addition, blocking with unlabeled compounds significantly reduced uptake into both the patient-derived R132H mutant and knockout cell lines [61]. Considering that the mutant enzyme was likely overexpressed in the transfected cell line (as indicated by much higher absolute uptake values compared to the patient-derived cell line), the smaller difference observed for the patient-derived cell line could point to appreciable binding of the radioligand to the wildtype enzyme. The latter would be consistent with the relatively low selectivity of AG-881 and analogs described above. Ex vivo biodistribution studies and in vivo PET imaging in a subcutaneous tumor xenograft model with the IDH1_R132H_-transfected glioma cell line showed good tumor uptake of the radioligand, with a tumor-to-brain ratio of 6.6 after 1 h, which was significantly reduced to 2.8 after pre-treatment with the non-labeled compound [61]. However, there was also significant uptake of radioactivity into bone that increased over time, suggesting extensive defluorination of the radioligand in vivo. Furthermore, the interpretation of the results is complicated by the fact that a subcutaneous (rather than an intracerebral) tumor model with the transfected (rather than the patient-derived) cell line was used for the ex vivo and in vivo studies. As such, the high tumor-to-brain ratios could at least in part reflect overexpression of IDH1_R132H_ in the tumor cells and/or limited brain uptake of the radioligand. Accordingly, similar tumor-to-brain ratios might not be achieved at clinically relevant enzyme concentrations in brain tumors harboring a native mutation. Nevertheless, the overall results of this first study with an aminotriazine-based radioligand encourage further exploration of this class with the aim of identifying tracers with improved metabolic stability and preferably selectivity for future studies. Starting points for the development of triazine-based tracers for glioma imaging could be AG-881 or one of the other fluorine-containing analogs, some of which have been shown to exhibit comparable (e.g., AGI-15056) or significantly better (e.g., AGI-12026) brain penetration (Table 1) [87]. In principle, radiofluorination of all these compounds could be achieved without structural modification by introducing fluorine-18 into one of the CF_3_ groups using known radiolabeling protocols (for a recent review, see [111]). In addition, the substitution of the aliphatic CF_3_ side-chains in AG-881 by various alkyl, cycloalkyl or aryl substituents has been shown to be possible with good retention of inhibitory potency (Table 5) [87]. Accordingly, radiolabeling without negative influence on the pharmacological properties could most likely also be achieved by replacing one of the side-chains with an ^18^F-labeled alkyl, aryl or heteroaryl scaffold. Such radioligands could usually be prepared in much higher molar activities than compounds containing an ^18^F-labeled CF_3_ group [111]. Alternatively, the 2-chloro-6-pyridyl or 2-CF_3_-6-pyridyl subunit in AG-881 or its analogs could be replaced with an ^18^F-labeled 2-fluoro-6-pyridyl subunit, although this could potentially reduce binding by disrupting the interaction with Asp_237_/Asp_312_ (Figure 12B). In the latter case, fluorine-18 could instead be introduced at another position of the pyridine ring (e.g., via ^18^F-fluorination of the appropriate iodonium salts, see [112,113]) while retaining the Cl or CF_3_ substituent at position 2 to maintain the formation of a halogen bond with Asp_237_/Asp_312_.

## 8. Summary and Concluding Remarks

In summary, mIDH-selective PET tracers could be relevant for several preclinical and clinical applications, which include non-invasive assessment of the IDH status and tumor delineation in glioma patients, longitudinal studies on mIDH expression in preclinical and clinical settings as well as in vivo target engagement and occupancy studies with mIDH-targeted drugs. However, only a few mIDH-selective radioligands have been evaluated so far, and most or all of them are unlikely to be suitable for glioma imaging, either because of limited brain penetration or due to other factors such as low metabolic stability, insufficient affinity and/or high non-specific binding. Further structural modification may be a viable approach to overcome these problems in some cases (e.g., aminotriazine-based radioligands), whereas in others (e.g., phenylglycine- or butylphenyl sulfonamide-based radioligands), it seems unlikely that further optimization will lead to promising candidates for glioma imaging. However, a number of additional mIDH inhibitors are available as potential leads, and some of them may represent promising starting points for tracer development. In particular, based on their ability to penetrate the BBB and other favorable PK properties, the most suitable leads apart from aminotriazine-based inhibitors such as AG-881 appear to be compounds based on a pyrimidinyl-oxazolidinone scaffold (such as the clinical candidate IDH305 and its preclinical analogs), an aminobenzimidazole scaffold (such as the clinical candidate BAY1436032), or a quinolinone scaffold (such as the clinical candidate FT2102 and its preclinical analogs). While tracers derived from aminotriazine-based inhibitors would have the advantage of being applicable for the detection of mIDH1 and mIDH2, their relatively low selectivity for mutant over wildtype enzymes is a potential disadvantage that could negatively affect the tumor-to-background ratios. In addition, recent studies indicate that the IDH1 wildtype enzyme may be overexpressed in over 60% of glioblastomas [114,115], suggesting that PET tracers with limited selectivity for mutated isoforms could give false positive results when used to assess the IDH status in these patients. For most other inhibitor classes with the potential for tracer development, excellent selectivity for the mutant enzymes has been demonstrated in functional assays, but it remains unclear to what extent these results correlate with the selectivity of binding in vivo. Additional factors that are difficult to predict based on the available data and could hamper development of mIDH-selective PET tracers are high non-specific binding to brain tissue and slow brain penetration or formation of (brain-penetrating) radiometabolites. Therefore, although the general properties of certain mIDH1 inhibitors appear to be suitable for tracer development and some of the preliminary results from studies with radiolabeled analogs are encouraging, it remains to be firmly established whether the development of mIDH-selective PET tracers from existing inhibitors is feasible.

## Figures and Tables

**Table 1:** Biochemical, cellular and pharmacokinetic (PK) properties of mIDH-selective inhibitors.

Compound	Biochemical IC_50_	Cellular IC_50_	Preclinical PK Properties
ML309 [60,70]	IDH1_R132H_: 96–335 nm IDH1_R132C_: 62–622 nm ^a^ IDH1_WT_: 21–36 µm IDH2_R172Q_: >30 µm IDH2_WT_: >30 µm	IDH1_R132H_: 150–248 nm IDH1_R132S_: 970 nm IDH1_R132G_: 711 nm IDH1_R132C_: 541–623 nm	- microsomal t_1/2_: 1–3 min - PAMPA P_app_: >170 × 10^−5^ cm/s - ER_Caco-2_: 2.9 - f_u,plasma_: 0.01 - plasma t_1/2_: >3 h - no brain penetration
AG-135 [60,65]	IDH1_R132H_: 42–375 nm IDH1_R132H/WT_: 80 nm ^b^ IDH1_R132C_: 4–182 nm ^a^ IDH1_WT_: 2–15 µm IDH2_R172K_: >10 µm IDH2_R172Q_: >30 µm IDH2_R140Q_: >10 µm IDH2_WT_: >10–30 µm	IDH1_R132H_: 81–217 nm IDH1_R132S_: 810 nm IDH1_R132G_: 681 nm IDH1_R132C_: 480–530 nm	- microsomal t_1/2_: 1.5–2.7 min - PAMPA P_app_: 79 × 10^−5^ cm/s - ER_Caco-2_: 13 - f_u,plasma_: 0.01
AGI-5198 [60,64,71,72,73,74]	IDH1_R132H_: 17–385 nm IDH1_R132C_: 0.2–13.3 µm ^a^ IDH1_WT_: >30–100 µm IDH2_R140Q_: >100 µm IDH2_R172Q_: >30 µm IDH2_R172K_: >100 µm IDH2_WT_: >30–100 µm	IDH1_R132H_: 43–70 nm IDH1_R132S_: 2 µm IDH1_R132G_: 1.6 µm IDH1_R132C_: 0.5–1.5 µm	- microsomal t_1/2_: 3–4 min - PAMPA P_app_: >140 × 10^−5^ cm/s - ER_Caco-2_: 23.1 - f_u,plasma_: 0.03–0.04 - no brain penetration
AG-120 (Ivosidenib) [60,73,75]	IDH1_R132H_: 12–40 nm IDH1_R132H/WT_: 5–12 nm ^b^ IDH1_R132C_: 13–205 nm ^a^ IDH1_R132G_: 8 nm IDH1_R132L_: 13 nm IDH1_R132S_: 12 nm IDH1_WT_: 0.024–4.3 µm IDH2_R172Q_: >30 µm IDH2_WT_: >30 µm	IDH1_R132H_: 19–50 nm IDH1_R132S_: 12–220 nm IDH1_R132G_: 16 nm IDH1_R132C_: 8–46 nm	- microsomal t_1/2_: >120 min - Cl_int(in vivo)_: 9 mL/min/kg - PAMPA P_app_: 54 × 10^−5^ cm/s - ER_Caco-2_: 2.0–56.4 - ER_MDR1-MDCK_: >350 - f_u,plasma_: 0.09 - K_p,brain_: 0.04 - plasma t_1/2_: 2.5–19 h
IDH889 [76]	IDH1_R132H_: 20 nm IDH1_R132C_: 72 nm IDH1_WT_: 1.38 µm	IDH1_R132H_: 14 nm	- Cl_int(in vitro)_: 143–588 µL/min/mg - f_u,plasma_: 0.01–0.03 - K_p,brain_: 1.4
IDH305 [75,77,78]	IDH1_R132H_: 27–50 nm IDH1_R132C_: 28–50 nm IDH1_WT_: 6.14 µm	IDH1_R132H_: 24 nm IDH1_R132C_: 53 nm IDH2_R140Q_: 3.8 µm IDH2_R172K_: 10 µm	- Cl_int(in vitro)_: 28–61 µL/min/mg - Cl_int(in vivo)_: 24–34 mL/min/kg - ER_Caco-2_: 1.2 - f_u,plasma_: 0.11–0.17 - f_u,brain_: 0.05–0.07 - K_p,brain_: 0.29–0.61 - K_p,uu,brain_: 0.17–0.18
Novartis 224 [60,79]	IDH1_R132H_: 17–130 nm IDH1_R132C_: 84–552 nm ^a^ IDH1_WT_: 3.9 µm IDH2_R172Q_: >30 µm IDH2_WT_: >30 µm	IDH1_R132H_: 52–92 nm IDH1_R132S_: 221 nm IDH1_R132G_: 121 nm IDH1_R132C_: 83–195 nm	- microsomal t_1/2_: 1–2 min - PAMPA P_app_: 156 × 10^−5^ cm/s - ER_Caco-2_: 1.7 - f_u,plasma_: 0.01
Novartis 530 [60,79]	IDH1_R132H_: 8.3–51 nm IDH1_R132C_: 32–98 nm ^a^ IDH1_WT_: 3.5 µm IDH2_R172Q_: >30 µm IDH2_WT_: >30 µm	IDH1_R132H_: 34–54 nm IDH1_R132S_: 78 nm IDH1_R132G_: 76 nm IDH1_R132C_: 49–52 nm	- microsomal t_1/2_: 1–3 min - PAMPA P_app_: 98 × 10^−5^ cm/s - ER_Caco-2_: 1.1 - f_u,plasma_: 0.02
Novartis 556 [60,79]	IDH1_R132H_: <72–141 nm IDH1_R132C_: 189–875 nm ^a^ IDH1_WT_: 10.5 µm IDH2_R172Q_: >30 µm IDH2_WT_: >30 µm	IDH1_R132H_: 186–334 nm IDH1_R132S_: 912 nm IDH1_R132G_: 1.1 µm IDH1_R132C_: 582 nm IDH1_R132C_: 686 nm	- microsomal t_1/2_: 8–24 min - PAMPA P_app_: 39 × 10^−5^ cm/s - ER_Caco-2_: 2.6 - f_u,plasma_: 0.14
2 (see Section 7.2) [75]	IDH1_R132H_: 4.0 nm IDH1_R132C_: 8.2 nm	IDH1_R132C_: 15.9 nm	- Cl_int(in vivo)_: 23 mL/min/kg - plasma t_1/2_: 1.5–2.5 h
BAY1436032 [44,80,81,82]	IDH1_R132H_: 15 nm IDH1_R132C_: 15 nm IDH1_WT_: 20 µm IDH2_WT_: >100 µm	IDH1_R132H_: 5–73 nm IDH1_R132C_: 5–135 nm IDH1_R132G_: 4 nm IDH1_R132L_: 3 nm IDH1_R132S_: 16 nm	- Cl_int(in vivo)_: 2.5 mL/min/kg - plasma t_1/2_: 3.1 h - K_p,brain_: 0.08–0.38 - ER_Caco-2_: 0.17
4 (see Section 7.4) [83]	IDH1_R132H_: 127 nm IDH1_R132C_: 2.25 µm IDH1_WT_: 100 µm	IDH1_R132H_: 266–316 nm IDH1_R132C_: 1.2–1.9 µm	- microsomal t_1/2_: <30 min - plasma t_1/2_: 1.27 h
5 (see Section 7.4) [83]	IDH1_R132H_: 18 nm IDH1_R132C_: 130 nm IDH1_WT_: 35 µm IDH2_R140Q_: 76.6 µm IDH2_R172K_: 33.8 µm	IDH1_R132H_: 18–45 nm IDH1_R132C_: 130–233 nm IDH1_R132G_: 120 nm IDH1_R132L_: 60 nm IDH1_R132S_: 1.5 µm	- Cl_int(in vitro)_: 7.0 µL/min/mg - Cl_int(in vivo)_: 3.3–8.6 mL/min/kg - plasma t_1/2_: 2.2–10.0 h - PAMPA P_app_: 180 × 10^−5^ cm/s - ER_Caco-2_: 1.0 - ER_MDR1-MDCK_: 1.19 - f_u,plasma_: 0.02–0.04
6 (see Section 7.4) [83]	IDH1_R132H_: 9 nm IDH1_R132C_: 36 nm	IDH1_R132H_: 1–11 nm IDH1_R132C_: 4–40 nm IDH1_R132G_: 3 nm IDH1_R132L_: 5 nm IDH1_R132S_: 129 nm	- Cl_int(in vitro)_: 7.0–10.1 µL/min/mg - Cl_int(in vivo)_: 4.3–16.4 mL/min/kg - plasma t_1/2_: 2.6–6.2 h - PAMPA P_app_: 127 × 10^−5^ cm/s - ER_Caco-2_: 1.65 - f_u,plasma_: 0.01–0.02
FT-2102 (Olutasidenib) [84,85]	IDH1_R132H_: 4.9–21 nm IDH1_R132C_: 114–178 nm IDH1_WT_: 22.4–>100 µm IDH2_R140Q_: >100 µm IDH2_R172K_: 27.3 µm IDH2_WT_: >100 µm	IDH1_R132H_: 9–21 nm IDH1_R132C_: 39–94 nm IDH1_R132L_: 42 nm IDH1_R132G_: 6 nm IDH1_R132S_: 9 nm	- PAMPA P_app_: 199 × 10^−5^ cm/s - ER_Caco-2_: 1.35 - K_p,brain_: 0.24–0.38
GSK321 [86]	IDH1_R132H_: 4.6 nm IDH1_R132C_: 3.8 nm IDH1_R132G_: 2.9 nm IDH1_WT_: 46 nm IDH2_R140Q_: 1.4 µm IDH2_R172S_: 1.0 µm IDH2_WT_: 496 nm	IDH1_R132C_: 85 nm	- ER_Caco-2_: 1.4
GSK864 [60,86]	IDH1_R132H_: 15–162 nm IDH1_R132C_: 8.8–668 nm ^a^ IDH1_R132G_: 16.6 nm IDH1_WT_: 0.5–2.7 µm IDH2_R140Q_: 1.9 µm IDH2_R172Q_: 22 nm IDH2_R172S_: 997 nm IDH2_WT_: >30 µm	IDH1_R132H_: 120–191 nm IDH1_R132S_: 532 nm IDH1_R132G_: 519 nm IDH1_R132C_: 299–341 nm	- microsomal t_1/2_: 13–73 min - PAMPA P_app_: 36 × 10^−5^ cm/s - ER_Caco-2_: 1.3–5.1 - f_u,plasma_: 0.01
AG-221 (Enasidenib) [60,87,88]	IDH1_R132H_: 5–>30 µm IDH1_R132H/WT_: 677 nm ^b^ IDH1_R132C_: 13–>30 µm ^a^ IDH1_WT_: 0.5–15.1 µm IDH2_R140Q_: 9–100 nm IDH2_R140Q/WT_: 40–380 nm ^b^ IDH2_R172Q_: 44 nm IDH2_R172K_: 200–400 nm IDH2_R172K/WT_: 30–180 nm ^b^ IDH2_WT_: 18–>30 µm	IDH2_R140Q_: 10–20 nm IDH2_R172K_: 0.5–1.6 µm	- microsomal t_1/2_: >120 min - Cl_int(in vivo)_: 13.8 mL/min/kg - PAMPA P_app_: 131 × 10^−5^ cm/s - plasma t_1/2_: 5.4 h - ER_Caco-2_: 2.5 - f_u,plasma_: 0.01 - K_p,brain_: 0.14
IDH2-C100 [60,88]	IDH1_R132H_: 9.4 µm IDH1_R132C_: 16–>30 µm ^a^ IDH1_WT_: >30 µm IDH2_R140Q_: 7 nm IDH2_R172Q_: 343 nm IDH2_WT_: 6.6 µm	IDH2_R140Q_: 30 nm	- microsomal t_1/2_: 13–27 min - PAMPA P_app_: 48 × 10^−5^ cm/s - ER_Caco-2_: 4.1 - f_u,plasma_: <0.01
AG-881 (Vorasidenib) [87]	IDH1_R132H_: 6–8 nm IDH1_R132H/WT_: 0.6–4 nm ^b^ IDH1_R132C_: 19 nm IDH1_R132G_: 17 nm IDH1_R132L_: 34 nm IDH1_R132S_: 6 nm IDH1_WT_: 4–190 nm IDH2_R140Q_: 12–118 nm IDH2_R140Q/WT_: 32–251 nm ^b^ IDH2_R172K_: 32–94 nm IDH2_R172K/WT_: 8–49 nm ^b^ IDH2_WT_: 31–374 nm	IDH1_R132H_: 3–3.2 nm IDH1_R132C_: 3.8–22 nm IDH1_R132S_: 0.8 nm IDH1_R132G_: 6.6 nm IDH2_R140Q_: 7.1–14 nm IDH2_R172K_: 130 nm	- K_p,brain_: 0.62–1.96
AGI-12026 [87]	IDH1_R132H_: 78 nm IDH1_R132H/WT_: 20 nm ^b^ IDH2_R140Q_: 19 nm		- K_p,brain_: 2.5
AGI-15056 [87]	IDH1_R132H_: 48 nm IDH1_R132H/WT_: 6 nm ^b^ IDH2_R140Q_: 22 nm	IDH1_R132H_: 2 nm IDH2_R140Q_: 14 nm	- K_p,brain_: 1.5

^a^ Upper limit determined with very high (5 mm) α-KG concentration; ^b^ determined with the respective mutant and wildtype IDH heterodimers. Abbreviations: microsomal t_1/2_—in vitro metabolic stability determined as half-life in liver microsomes; Cl_int(in vitro)_—in vitro metabolic stability determined as intrinsic clearance in hepatocytes or liver microsomes; Cl_int(in vivo)_—in vivo metabolic stability determined as intrinsic clearance in pharmacokinetic studies; plasma t_1/2_—in vivo half-life in blood determined in pharmacokinetic studies; PAMPA P_app_—apparent permeability coefficient determined in parallel artificial membrane permeability assays (PAMPA); ER_Caco-2_—efflux ratio determined in Caco-2 cells; ER_MDR1-MDCK_—efflux ratio determined in MDCK cells transfected with the efflux transporter P-gp; f_u,plasma_—unbound fraction of drug in plasma; f_u,brain_—unbound fraction of drug in brain; K_p,brain_—concentration ratio of total drug in brain and blood; K_p,uu,brain_—concentration ratio of unbound drug in brain and blood.

**Table 2:** Pyrimidinyl-oxazolidinone-based mIDH1-inhibitors listed by decreasing potency.

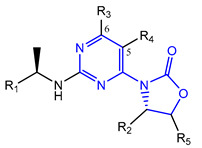					
R_1_	R_2_	R_3_	R_4_	R_5_	IC_50_^a^
** 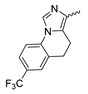 **	** 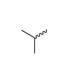 **	H	H	H	1 nm
** 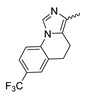 **	** 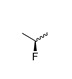 **	H Cl	F H	H H	2 nm 2 nm
** 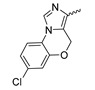 **	** 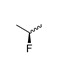 **	H	H	H	2 nm
** 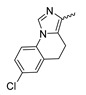 **	** 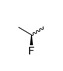 **	H	H	H	2 nm
** 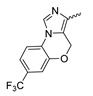 **	** 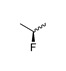 **	H	H	H	3 nm
** 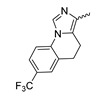 **	** 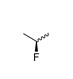 **	H	H	H	4 nm ^b^
** 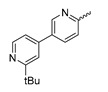 **	** 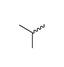 **	H	H	H	4 nm
** 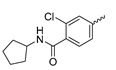 **	** 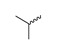 **	H	H	H	8 nm ^c^
** 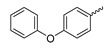 **	** 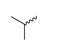 **	H	H	H	10 nm
** 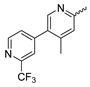 **	** 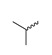 **	H	H	H	13 nm
** 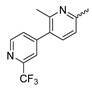 **	** 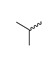 **	H	H	H	15 nm
** 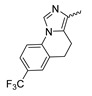 **	** 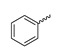 **	H	H	H	15 nm
** 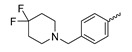 **	** 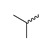 **	H	H	H	17 nm ^d^
** 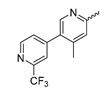 **	** 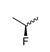 **	H	H	H	18 nm ^e^
** 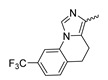 **	** 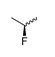 **	H	H	H	18 nm
** 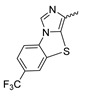 **	** 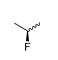 **	H	H	H	19 nm
** 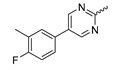 **	** 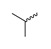 **	H	H	H	20 nm ^f^
** 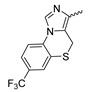 **	** 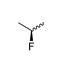 **	H	H	H	24 nm
** 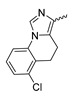 **	** 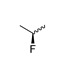 **	H	H	H	24 nm
** 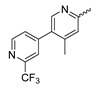 **	** 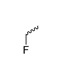 **	H	H	H	28 nm
** 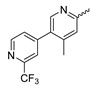 **	** 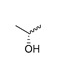 **	H	H	H	33 nm
** 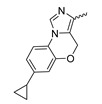 **	** 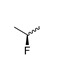 **	H	H	H	33 nm
** 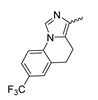 **	** 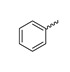 **	H	H	** 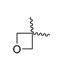 **	34 nm
** 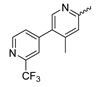 **	** 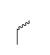 **	H	H	H	35 nm
** 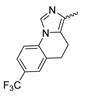 **	** 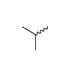 **	H	H	** 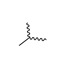 **	42 nm
** 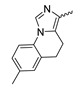 **	** 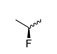 **	H	H	H	43 nm
** 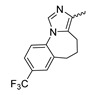 **	** 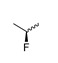 **	H	H	H	44 nm
** 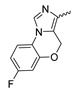 **	** 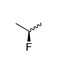 **	H	H	H	48 nm
** 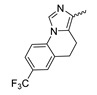 **	** 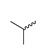 **	H	H	** 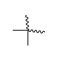 **	51 nm
** 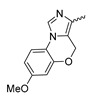 **	** 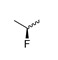 **	H	H	H	62 nm
** 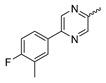 **	** 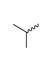 **	H	H	H	62 nm
** 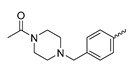 **	** 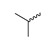 **	H	H	H	72 nm ^g^
** 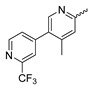 **	** 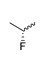 **	H	H	H	73 nm
** 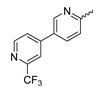 **	** 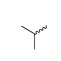 **	H	H	H	77 nm
** 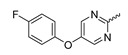 **	** 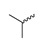 **	H	H	H	81 nm
** 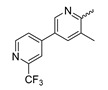 **	** 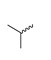 **	H	H	H	84 nm
** 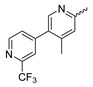 **	Me	H	H	H	116 nm
** 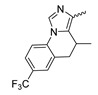 **	** 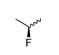 **	H	H	H	120 nm
** 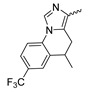 **	** 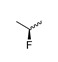 **	H	H	H	128 nm
** 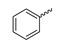 **	** 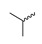 **	H	H	H	220 nm
** 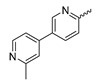 **	** 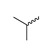 **	H	H	H	339 nm
** 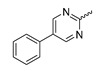 **	** 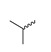 **	H	H	H	340 nm
** 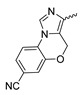 **	** 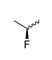 **	H	H	H	478 nm
** 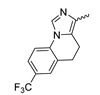 **	H	H	H	** 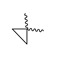 **	>10,000 nm

^a^ IC_50_ values for biochemical assays with IDH1_R132H_ homodimers, compiled from [76,77,79,97]; ^b^ **2**; ^c^ Novartis 530; ^d^ Novartis 224; ^e^ IDH305; ^f^ IDH889; ^g^ Novartis 556.

**Table 3:** Aminobenzimidazole-based mIDH1-inhibitors listed by decreasing potency.

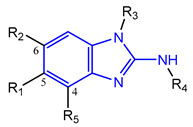					
R_1_	R_2_	R_3_	R_4_	R_5_	IC_50_^a^
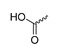	OMe	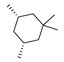	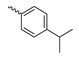	H	3 nm
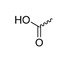	H	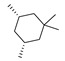	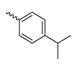	H	4 nm
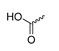	Me	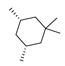	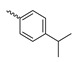	H	4 nm
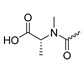	Cl	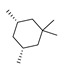	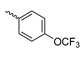	H	6 nm
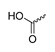	OMe	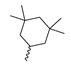	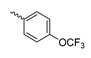	H	6 nm
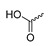	Me	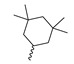	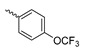	H	7 nm
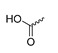	OMe	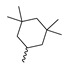	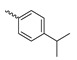	H	8 nm
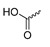	H	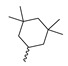	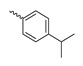	F	9 nm
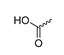	OMe	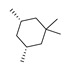	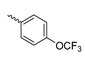	H	9 nm
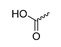	OMe	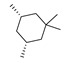	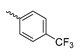	H	9 nm
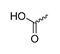	H	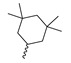	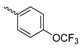	H	10 nm
H	OEt	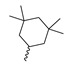	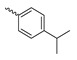	H	10 nm
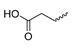	H	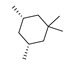	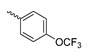	H	15 nm ^b^
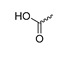	Me	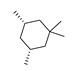	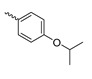	H	20 nm
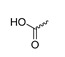	OMe	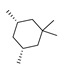	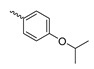	H	20 nm
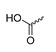	Me	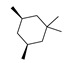	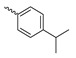	H	20 nm
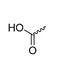	H	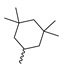	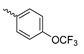	F	20 nm
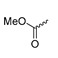	Me	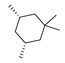	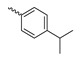	H	20 nm
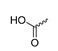	H	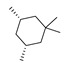	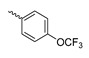	H	20 nm
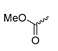	OMe	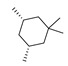	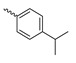	H	20 nm
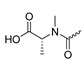	F	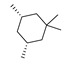	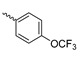	H	20 nm
H	Me	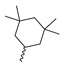	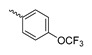	H	20 nm
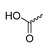	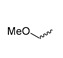	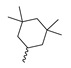	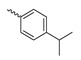	H	20 nm
H	Me	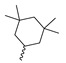	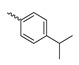	F	20 nm
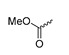	OMe	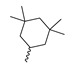	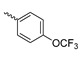	H	30 nm
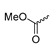	Me	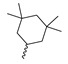	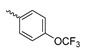	H	30 nm
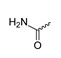	Me	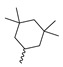	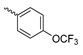	H	30 nm
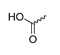	H	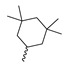	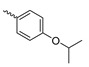	F	30 nm
H	H	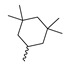	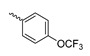	H	30 nm
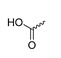	H	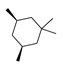	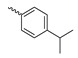	H	30 nm
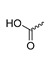	Cl	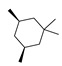	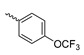	H	30 nm
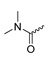	H	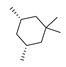	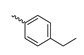	H	30 nm
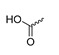	OMe	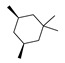	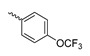	H	30 nm
OMe	H	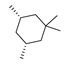	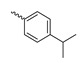	H	30 nm
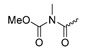	Cl	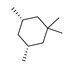	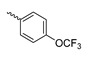	H	30 nm
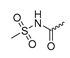	Me	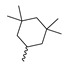	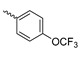	H	30 nm
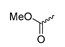	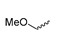	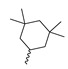	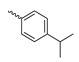	H	30 nm
H	H	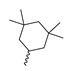	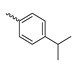	OMe	30 nm
H	Me	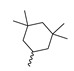	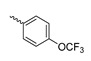	Me	40 nm
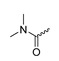	H	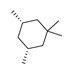	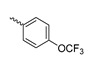	H	40 nm
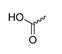	Me	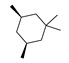	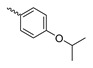	H	40 nm
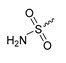	H	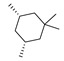	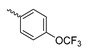	H	40 nm
H	OEt	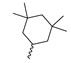	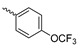	H	40 nm
H	OMe	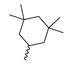	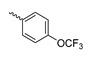	H	40 nm
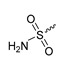	H	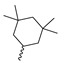	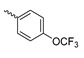	H	50 nm
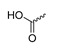	H	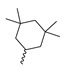	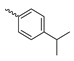	H	50 nm
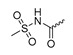	Cl	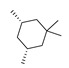	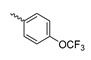	H	60 nm
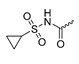	Me	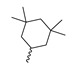	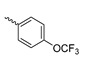	H	60 nm
H	OMe	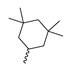	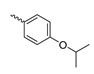	H	60 nm
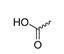	H	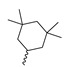	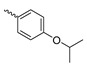	OMe	70 nm
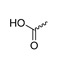	H	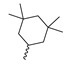	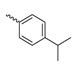	OMe	70 nm
H	H	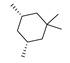	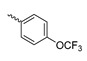	H	70 nm
H	H	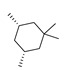	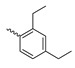	F	70 nm
H	H	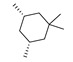	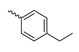	F	70 nm
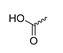	H	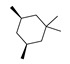	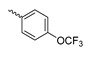	H	70 nm
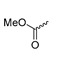	OMe	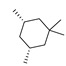	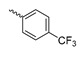	H	70 nm
H_2_N	H	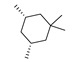	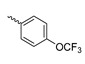	H	70 nm
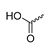	H	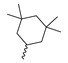	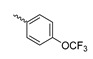	OMe	70 nm
OMe	H	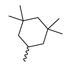	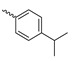	H	70 nm
H	Me	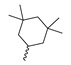	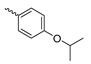	F	70 nm
H_2_N	H	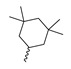	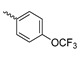	H	80 nm
CN	H	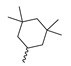	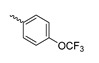	H	80 nm
CN	Me	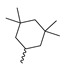	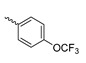	H	80 nm
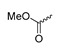	OMe	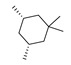	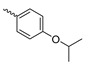	H	80 nm
H	H	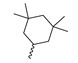	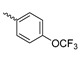	OMe	80 nm
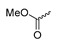	H	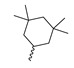	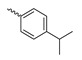	H	90 nm
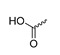	H	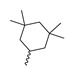	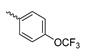	Me	90 nm
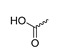	Cl	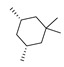	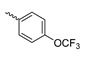	H	90 nm
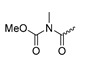	Cl	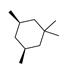	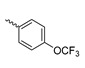	H	90 nm
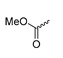	OMe	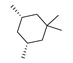	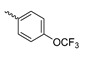	H	90 nm
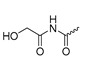	H	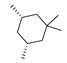	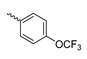	H	90 nm
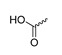	H	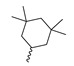	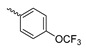	OMe	100 nm

^a^ IC_50_ values for biochemical assays with IDH1_R132H_ homodimers, compiled from [80,98]; ^b^ BAY1436032.

**Table 4:** Quinolinone-based mIDH1-inhibitors listed by decreasing potency.

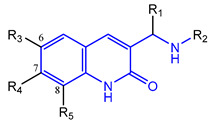					
R_1_	R_2_	R_3_	R_4_	R_5_	IC_50_^a^
(*S*)-Me	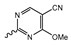	Cl	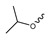	H	4 nm
(*S*)-Me	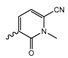	I	H	H	5 nm ^b^
H	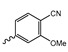	Cl	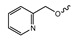	H	6 nm
(*S*)-Me	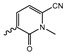	Cl	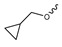	H	8 nm
(*S*)-Me	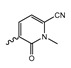	Cl	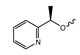	H	8 nm
(*S*)-Me	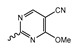	Cl	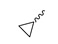	H	9 nm
(*S*)-Me	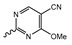	Cl	OMe	H	9 nm ^c^
(*S*)-Me	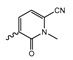	Cl	OMe	H	11 nm
(*S*)-Me	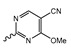	Cl	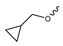	H	11 nm
(*S*)-Me	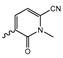	Cl	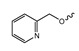	H	11 nm
H	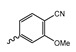	Cl	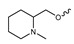	H	13 nm
(*S*)-Me	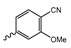	Cl	H	H	14 nm
H	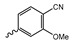	Cl	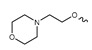	H	16 nm
(*S*)-Me	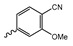	Cl	H	H	17 nm
(*S*)-Me	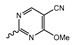	Cl	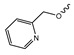	H	17 nm
(*S*)-Me	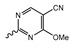	Cl	H	H	18 nm ^d^
(*S*)-Me	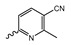	Cl	H	H	20 nm
(*S*)-Me	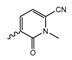	Cl	H	H	21 nm ^e^
H	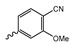	Cl	H	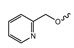	22 nm
H	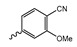	Cl	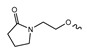	H	23 nm
(*S*)-Me	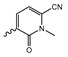	F	H	H	23 nm ^f^
(*S*)-Me	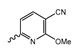	Cl	H	H	24 nm
(*S*)-Me	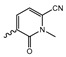	Cl	F	H	25 nm
H	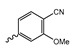	Cl	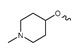	H	32 nm
(*S*)-Me	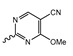	Cl	F	H	36 nm
H	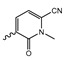	Cl	H	H	40 nm
(*S*)-Me	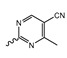	Cl	H	H	40 nm
(*S*)-Me	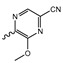	Cl	H	H	44 nm
H	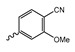	CF_3_	H	H	53 nm
H	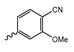	Br	H	H	54 nm
H	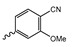	Cl	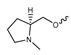	H	56 nm
H	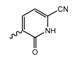	Cl	H	H	65 nm
H	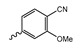	Cl	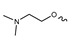	H	69 nm
H	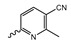	Cl	H	H	72 nm
H	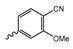	*t*Bu	H	H	76 nm
H	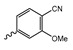	Cl	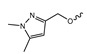	H	77 nm
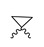	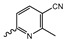	Cl	H	H	100 nm
H	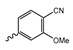	Cl	H	H	127 nm ^g^
H	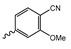	Cl	OMe	H	127 nm
H	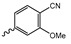	Me	H	H	132 nm
H	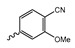	F	H	H	138 nm
H	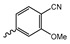	OMe	H	H	140 nm
H	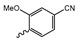	Cl	H	H	147 nm
*rac*-Me	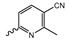	Cl	H	H	147 nm
H	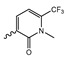	Cl	H	H	160 nm
H	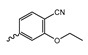	Cl	H	H	188 nm
H	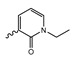	Cl	H	H	223 nm
H	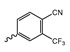	Cl	H	H	231 nm
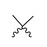	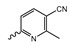	Cl	H	H	295 nm
(*R*)-Me	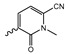	I	H	H	323 nm
H	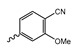	Cl	H	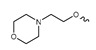	341 nm
H	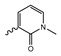	Cl	H	H	358 nm
H	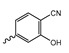	Cl	H	H	382 nm
H	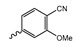	Cl	H	OMe	455 nm
H	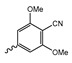	Cl	H	H	464 nm
H	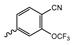	Cl	H	H	487 nm
H	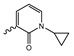	Cl	H	H	694 nm
(*R*)-Me	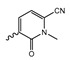	F	H	H	698 nm
H	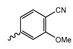	H	H	H	913 nm
H	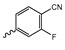	Cl	H	H	995 nm
H	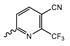	Cl	H	H	1260 nm
H	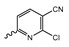	Cl	H	H	25,000 nm
(*R*)-Me	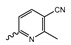	Cl	H	H	19,300 nm
H	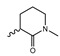	Cl	H	H	25,000 nm
H	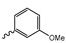	Cl	H	H	>25,000 nm

^a^ IC_50_ values for biochemical assays with IDH1_R132H_ homodimers, compiled from [83,84,85]; ^b^ **7**; ^c^ **6**; ^d^ **5**; ^e^ FT-2102 (Olutasidenib); ^f^ **8**; ^g^ **4**.

**Table 5:** Aminotriazine-based mIDH1/2-inhibitors listed by decreasing potency.

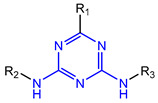			
R_1_	R_2_	R_3_	IC_50_^a^
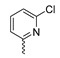	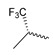	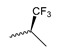	0.6 nm ^b^
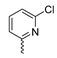	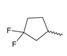	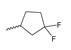	6 nm
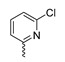	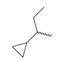	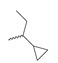	6 nm
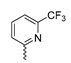	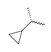	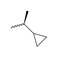	6 nm ^c^
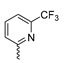	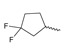	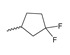	6 nm
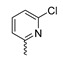	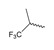	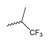	7 nm
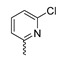	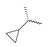	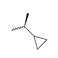	9 nm
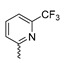	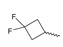	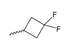	9 nm ^d^
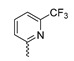	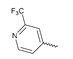	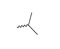	20 nm ^e^
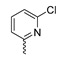	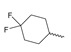	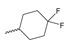	22 nm
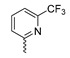	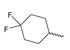	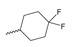	27 nm
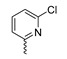	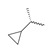	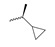	32 nm
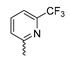	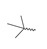	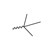	60 nm
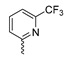	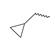	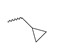	83 nm
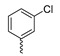	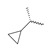	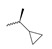	140 nm
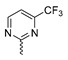	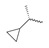	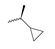	185 nm
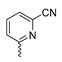	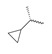	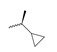	201 nm
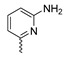	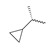	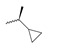	394 nm
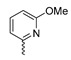	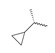	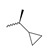	677 nm
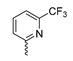	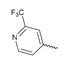	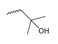	677 nm ^f^
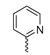	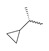	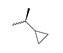	1566 nm
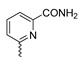	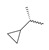	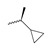	1749 nm
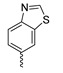	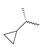	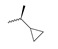	No fit
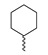	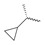	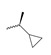	No fit

^a^ IC_50_ values for biochemical assays with IDH1_R132H/WT_ heterodimers, compiled from [87,88]; ^b^ AG-881 (Vorasidenib); ^c^ AGI-15056; ^d^ **12**; ^e^ AGI-12026; ^f^ AG-221 (Enasidenib).

## Data Availability

Not applicable.
